# KMT9 drives T cell exclusion and dysfunction by promoting PMN-MDSCs infiltration and ARG1 expression in prostate cancer

**DOI:** 10.1186/s12943-026-02801-8

**Published:** 2026-09-17

**Authors:** Jon Peñarando, Dominica Willmann, Manuela Sum, Yanhan Jia, Christopher Berlin, Lukas M. Braun, Zihao Chen, Sylvia Urban, Manfred Jung, Delphine Duteil, Daniel Metzger, Christian Gratzke, Robert Zeiser, Holger Greschik, Roland Schüle, Eric Metzger

**Affiliations:** 1 Klinik Für Urologie Und Zentrale Klinische Forschung, Klinikum Der Albert-Ludwigs-Universität Freiburg, Freiburg, Germany; 2 Klinik Für Allgemein- Und Viszeralchirurgie, Klinikum Der Albert-Ludwigs-Universität Freiburg, Freiburg, Germany; 3 Department of Internal Medicine I, Medical Center - University of Freiburg, Faculty of Medicine, University of Freiburg, Freiburg, Germany; 4 Institute of Pharmaceutical Sciences, Albert-Ludwigs-Universität Freiburg, Freiburg, Germany; 5 Institut de Génétique Et de Biologie Moléculaire Et Cellulaire, CNRS UMR7104, INSERM U964, Université de Strasbourg, Illkirch, France; 6 German Cancer Consortium (DKTK), Partner Site Freiburg, a partnership between DKFZ and Medical Center - University of Freiburg, Freiburg, Germany; 7 CIBSS Centre of Biological Signalling Studies, University of Freiburg, Freiburg, Germany

**Keywords:** KMT9, Histone methyltransferase, Tumour immune microenvironment, Immunosuppressive MDSCs, Prostate cancer

## Abstract

Immunotherapy has emerged as a revolutionary therapeutic approach to treat cancer showing remarkable clinical responses. However, its efficacy in solid tumours such as prostate cancer (PCa) remains very limited due to a highly immunosuppressive tumour immune microenvironment (TIME) that hampers cytotoxic T cell infiltration and activity. Here, we show that lysine methyltransferase 9 (KMT9) governs the formation of an immunosuppressive TIME in PCa. KMT9 regulates the expression of tumour-secreted myeloid-attracting C-X-C motif chemokine receptor 2 (CXCR2) ligands such as C-X-C motif chemokine ligand 5 (CXCL5) thereby promoting the infiltration of immunosuppressive polymorphonuclear myeloid-derived suppressor cells (PMN-MDSCs) within tumours. These changes collectively result in decreased activation and exclusion of cytotoxic T cells from tumour glands. Furthermore, we demonstrate that KMT9 confers tumour cell-resistance to T cell cytotoxicity by regulating expression of arginase 1 (ARG1). Accordingly, KMT9α ablation results in inhibition of prostate tumour growth accompanied by a massive reduction of PMN-MDSC recruitment and a significant increase in cytotoxic T cell activation and infiltration of the prostate tumour glands. Together, we uncovered KMT9 as a regulator of immune evasion that promotes an immune-excluded TIME in prostate tumours. Furthermore, our findings establish KMT9 as a therapeutic target to reprogram the immunosuppressive landscape and potentially improve the clinical efficacy of current immunotherapies.

**Graphical Abstract:**

**Figure:**
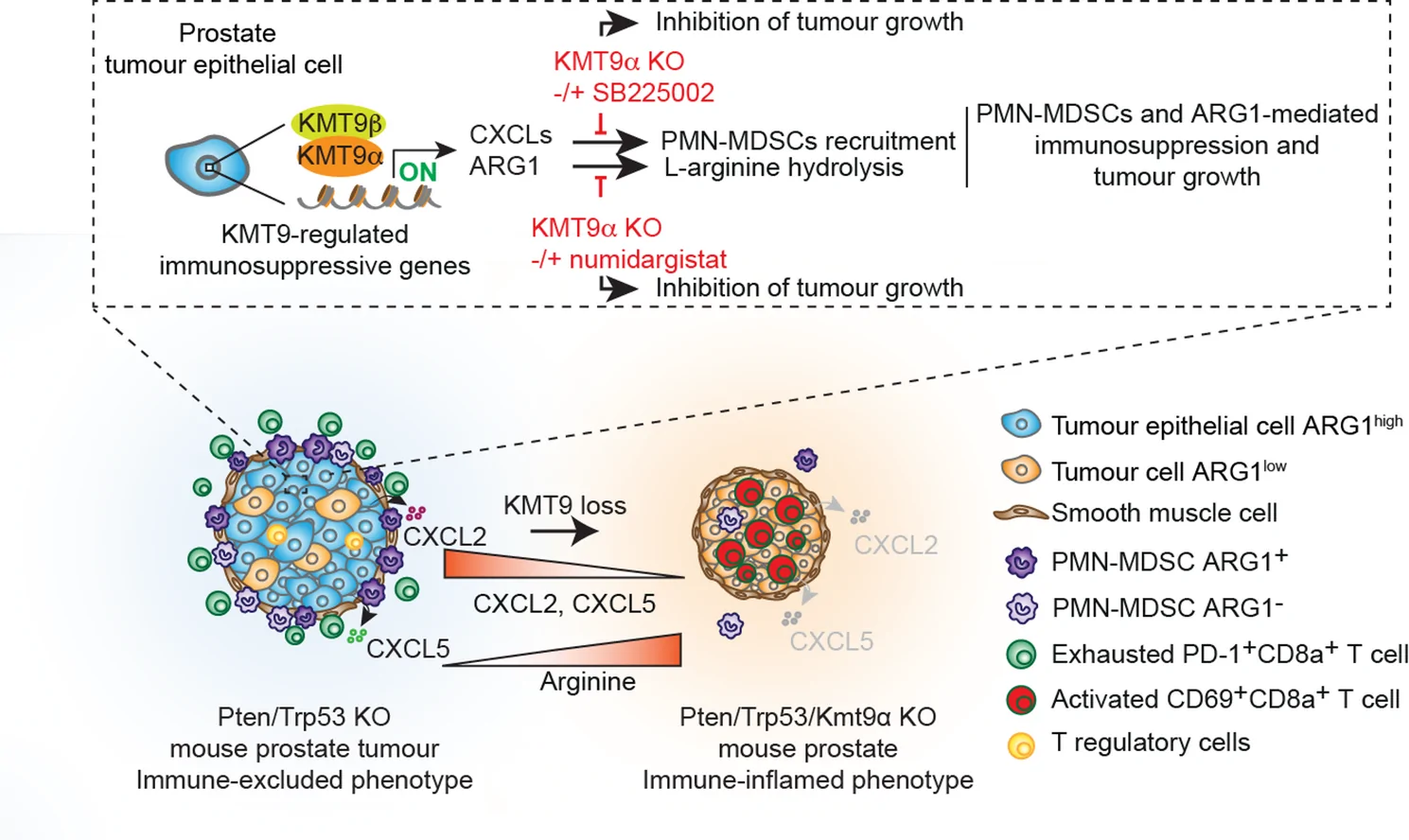

**Supplementary Information:**

The online version contains supplementary material available at https://doi.org/10.1186/s12943-026-02801-8.

## Introduction

Despite advances in androgen deprivation therapy, PCa remains a severe therapeutic challenge due to the development of treatment resistance [1]. Alternative treatment options such as immunotherapy show limited benefit in solid tumours like PCa due to a highly immunosuppressive TIME [2]. Solid tumours have been classified into three immune phenotypes based on T cell distribution within the TIME [3, 4]. The immune-inflamed phenotype is characterized by high infiltration of T cells compared to the absence of T cell infiltration in the immune-desert phenotype. Third, the immune-excluded phenotype is characterized by abundant infiltration of T cells which are, however, restricted to the surrounding stroma and unable to penetrate the tumour cell nests [3, 5, 6]. Distinction of these immune phenotypes is critical for successful immunotherapy since effective clinical responses require both proper T cell activation and direct contact with tumour cells [7, 8]. Notably, immune checkpoint inhibitors have been reported to activate T cells within the stroma but rarely elicit beneficial clinical responses in solid tumours [3, 4]. PCa is considered a “cold” tumour showing poor infiltration of lymphocytes, high presence of immunosuppressive cells and low numbers of tumour-associated antigens that hamper the effectiveness of immunotherapies [9]. Therefore, elucidating the underlying mechanisms responsible for T cell dysfunction and exclusion from tumour nests is key to the success of T cell-based immunotherapies.

PMN-MDSCs have emerged as major mediators of immunosuppression in most solid tumours including PCa [10–13]. PMN-MDSCs, together with tumour cells, mediate immunosuppression in part via arginase 1 (ARG1), an enzyme that catalyses the hydrolysis of L-arginine to urea and L-ornithine. The action of ARG1 depletes extracellular L-arginine and, in consequence, affects T-cell abundance, since T cells require L-arginine for proliferation [14]. Accordingly, targeting ARG1 is an emerging therapeutic approach for cancer treatment that could shift arginine metabolism to favour lymphocyte proliferation [15]. In addition, PMN-MDSCs have been reported to promote T cell exclusion from tumours by physically reducing contacts between T cells and tumour cells [16]. PMN-MDSCs are recruited to the TIME by C-X-C motif chemokine ligands (CXCL) that bind to and activate CXCR2 present on the cell surface. Targeting CXCR2 with a potent inhibitor has been explored as a means to prevent infiltration of the TIME by PMN-MDSCs [13, 17, 18]. In a recent study, concomitant treatment with AZD5069, a CXCR2 inhibitor, and enzalutamide (blocking androgen receptor (AR) signalling) resulted in durable clinical benefit in a subset of human patients with metastatic castration-resistant prostate cancer (mCRPC) [13]. Together, these studies underline the importance to gain further insight into the mechanisms governing PMN-MDSC recruitment and T cell activity to identify new avenues for T cell-based immunotherapy in PCa.

We previously identified KMT9 as a novel target for the treatment of PCa [19]. KMT9 is an obligatory heterodimer composed of the catalytic subunit KMT9α and the structural subunit KMT9β that monomethylates histone H4 at lysine 12 in addition to other non-histone targets [19–24]. In prostate and other tumours, KMT9 controls the transcription of target genes involved in cell cycle regulation thereby promoting tumour cell proliferation [19, 25, 26]. Depletion or enzymatic inactivation of KMT9 blocks proliferation of castration- and enzalutamide-resistant PCa cells independently of AR signalling in vitro and in a xenograft mouse model [19]. However, since in early studies we used PCa cell lines and xenografts in immunocompromised mice, it remained unclear whether KMT9 exerts additional immunomodulatory functions within the TIME of prostate tumours in vivo.

To evaluate the impact of *Kmt9α* ablation on the PCa TIME, we used the well-established *phosphatase and tensin homolog (Pten)/transformation related protein (Trp) 53* knockout (KO) mouse model [27, 28]. While *Pten/Trp53* KO mice developed tumours and an immunosuppressive TIME in accordance with previous reports [29, 30], we observed a strongly reduced tumour size and profound alterations of the TIME in *Pten/Trp53/Kmt9α* KO mice. We found that KMT9 upregulated the expression of myeloid-attracting chemokines such as CXCL5, causing PMN-MDSC recruitment to *Pten/Trp53* KO tumours. *Pten/Trp53* KO prostate tumour glands were found shielded by a barrier of peritumoral PMN-MDSCs, and cytotoxic T cell infiltration was restricted to the stroma. Furthermore, KMT9 regulated the expression of ARG1, controlling arginine levels and thereby promoting T cell exhaustion in *Pten/Trp53* KO prostate tumours. Due to downregulation of CXCL5 and ARG1 upon *Kmt9α* ablation, the TIME of *Pten/Trp53/Kmt9α* KO mice was characterized by a loss of the PMN-MDSC barrier and increased infiltration of tumour glands by activated cytotoxic T cells. These combined anti-tumour effects of *Kmt9α* ablation could be further enhanced by treatment of *Pten/Trp53/Kmt9α* KO mice with the CXCR2 inhibitor SB225002 or the ARG1 inhibitor numidargistat. Together, our study identifies KMT9 as an important regulator of immune evasion in PCa and pave the way for novel treatment options for the implementation of immunotherapies.

## Results

### KMT9 controls the expression of MDSC-chemoattractants in prostate cancer

**Fig. 1:**
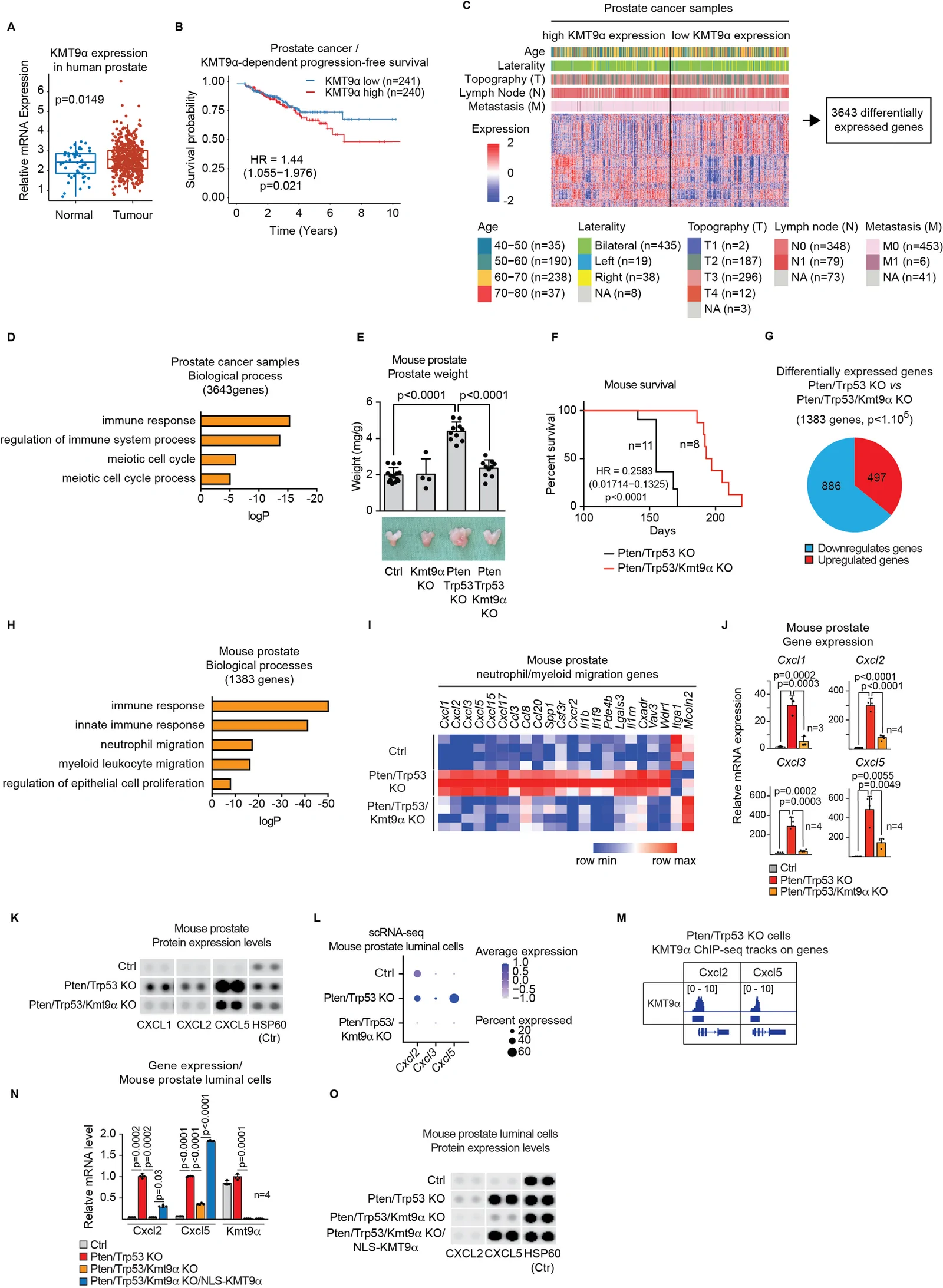
KMT9 controls expression of PMN-MDSC-chemoattractants in PCa. **A**, Box-Whisker plots with min to max values showing *Kmt9α* mRNA expression in healthy human prostate (*n* = 52) and prostate tumour samples (*n* = 500). **B**, Kaplan–Meier progression-free survival analysis for patients with prostate tumours expressing high (red, *n* = 240) or low (blue, *n* = 241) levels of *Kmt9α* mRNA. HR = hazard ratio. **C**, Heatmap displaying differential gene expression in human prostate tumours expressing high or low levels of *Kmt9α* mRNA. Samples were divided into high- and low- *Kmt9α* groups according to the median of *Kmt9α* expression values. **A**-**C**, Data were extracted from TCGA-PRAD. **D**, Enriched pathway analysis for 3643 genes differentially expressed in human prostate tumours expressing high or low levels of *Kmt9α* mRNA. Bars represent individual Benjamini logP values derived from GO enrichment analysis. **E**, Bar graph representing the prostate weight of Ctrl (*n* = 14), *Kmt9α* KO (*n* = 4), *Pten/Trp53* KO (*n* = 10), and *Pten/Trp53/Kmt9α* KO (n = 10) mice eight weeks after tamoxifen-induced gene deletion. A representative picture of the prostates is shown below the bar graph. **F,** Kaplan–Meier progression survival curve of *Pten/Trp53* KO (n = 11) and *Pten/Trp53/Kmt9α* KO (*n* = 8) mice. **G**, Diagram showing the number of genes differentially expressed between *Pten/Trp53* KO and *Pten/Trp53/Kmt9α* KO prostates. **H**, Enriched pathway analysis for 1383 genes differentially expressed between *Pten/Trp53* KO and *Pten/Trp53/Kmt9α* KO prostates. Bars represent individual Benjamini logP values derived from GO enrichment analysis. **I**, Heatmap displaying mRNA levels of differentially expressed genes involved in neutrophil/myeloid cell migration in Ctrl, *Pten/Trp53* KO, and *Pten/Trp53/Kmt9α* KO mice prostates. **J**, QRT-PCR analysis showing relative mRNA levels of *Cxcl1* (n = 3), *Cxcl2* (n = 4), *Cxcl3* (n = 3), and *Cxcl5* (n = 4) in Ctrl, *Pten/Trp53* KO, and *Pten/Trp53/Kmt9α* KO prostates. Data represent means + s.d. n represents the number of biologically independent samples. **K**, Analysis of protein expression of the indicated myeloid chemoattractants by Western blot of Ctrl, *Pten/Trp53* KO, and *Pten/Trp53/Kmt9α* KO mice prostates using a proteome chemokine array. **L**, Dot blot displaying single-cell mRNA expression analysis of Cxcl2, Cxcl3, and Cxcl5 in luminal cells of Ctrl, *Pten/Trp53* KO, and *Pten/Trp53/Kmt9α* KO prostates. **M**, ChIP-seq tracks showing KMT9α at representative genes in *Pten/Trp53* KO mouse prostate tumour cells. The experiment was repeated two times independently with similar results. **N**, QRT-PCR analysis showing relative mRNA levels of *Cxcl2*, *Cxcl5*, and *Kmt9α* (n = 4 biologically independent samples) in Ctrl, *Pten/Trp53* KO, *Pten/Trp53/Kmt9α* KO, and *Pten/Trp53/Kmt9α* KO/NLS-KMT9α mouse prostate luminal cells. Data represent means + s.d. **O**, Analysis of protein expression of the indicated myeloid chemoattractants by Western blot in Ctrl, *Pten/Trp53* KO, *Pten/Trp53/Kmt9α* KO, *Pten/Trp53/Kmt9α* KO/NLS-KMT9α mouse prostate luminal cells using a proteome chemokine array. P values obtained by unpaired Student T test (**A**) one-way ANOVA test (**E**,** J**,** N**) and Mantel Cox test (**F**)

To gain novel insight into KMT9 biology in PCa, we analysed the Cancer Genome Atlas Prostate Adenocarcinoma (TCGA-PRAD) cohort of 500 human PCa and 52 normal prostate samples (Supplemental Fig. 1A). Consistent with our previous data [19], *KMT9α* and *β* mRNA expression levels were increased in PCa compared with normal prostate (Fig. 1A and Supplemental Fig. 1B). Of note, high expression of either gene correlate with reduced progression-free survival (Fig. 1B and Supplemental Fig. 1B). We separated the PCa cohorts into high- and low-KMT9α groups and investigated their transcriptomes, which revealed 3643 differentially expressed genes (DEGs) (Fig. 1C). Enrichment analysis for biological processes uncovered not only genes responsible for meiotic cell cycle control but also gene sets involved in immune response and regulation of immune system processes among the top-ranking categories (Fig. 1D). These data suggest that KMT9 not only regulates tumour cell proliferation as previously shown [19] but might also regulate the immune response and TIME in vivo.

Our analyses also uncovered increased *KMT9α* expression in human PCa with *PTEN* deletion either alone or associated with mutation of *TP53* (Supplemental Fig. 1D). It has been previously reported that loss of PTEN and TP53 are key events driving TIME immunosuppression and immune evasion in PCa [2]. To interrogate potential functions of KMT9 in the PCa TIME, we investigated PCa progression in the presence or absence of KMT9α using the well-established *PSA-Cre-ER*^*T2(Tg/0)*^*/Pten*^*fl/fl*^*/Trp53*^*fl/fl*^ mouse PCa model [28]. Upon tamoxifen treatment, the combinatorial deletion of the tumour suppressors *Pten* and *Trp53* in prostate luminal cells (hereafter termed *Pten/Trp53* KO) induces highly aggressive PCa [27, 28]. We also mated *Kmt9α*^fl/fl^ with *PSA-Cre-ER*^*T2(Tg/0)*^ and *PSA-Cre-ER*^*T2(Tg/0)*^*/Pten*^*fl/fl*^*/Trp53*^*fl/fl*^ mice to generate *PSA-Cre-ER*^*T2(Tg/0)*^*/Kmt9α*^*fl/fl*^ and *PSA-Cre-ER*^*T2(Tg/0)*^*/Pten*^*fl/fl*^*/Trp53*^*fl/fl*^*/Kmt9α*^*fl/f*l^ mice. By treatment of ten weeks old mice with tamoxifen, we obtained prostate-specific *Kmt9α* KO and *Pten/Trp53/Kmt9α* KO mice. Tamoxifen-treated *PSA-Cre-ER*^*T2(Tg/0)*^ mice served as control (Ctrl). Ablation of *KMT9α* (*Kmt9α* KO) had no impact on prostate size compared with Ctrl prostates (Fig. 1E Supplemental Fig. 1E). Furthermore, a comparison of haematoxylin-stained *Kmt9*α KO with Ctrl prostates did not reveal obvious morphological changes (Supplemental Fig. 1F). Specific loss of KMT9α in luminal cells of the prostate was verified by immunohistochemistry and Western blot analysis (Supplemental Fig. 1G, H). As previously described, *Pten/Trp53* KO mice exhibited significantly enlarged prostates compared with Ctrl mice [27, 28] (Fig. 1E), and our analyses revealed the presence of tumours expressing elevated levels of KMT9α (Supplemental Fig. 1I, K, L). Importantly, the absence of KMT9α in *Pten/Trp53/Kmt9α* KO mice resulted in impaired tumour growth (Fig. 1E) and prolonged mice survival (Fig. 1F). We verified loss of both PTEN and TRP53 as well as activation of AKT signalling, a known consequence of *Pten* loss [27], in *Pten/Trp53* KO prostates (Supplemental Fig. 1I) and confirmed absence of KMT9α in epithelial cells of *Pten/Trp53/Kmt9α* KO prostates (Supplemental Fig. 1J-L).

To gain insights into KMT9-dependent mechanisms regulating prostate tumour growth, we performed a global transcriptome analysis (RNA-seq) with Ctrl, *Pten/Trp53* KO, and *Pten/Trp53/Kmt9α* KO prostates. Comparison of *Pten/Trp53/Kmt9α* KO with *Pten/Trp53* KO transcriptomes identified 1383 DEGs (Fig. 1G). Gene enrichment analyses for these DEGs uncovered biological processes including immune response, migration of neutrophils/myeloid cells, and regulation of epithelial cell proliferation among the top-ranking categories (Fig. 1H). The enriched gene sets included DEGs involved in migration of PMN-MDSCs such as *C-X-C motif chemokine ligands (Cxcl) 1, 2, 3, 5* which were upregulated in *Pten/Trp53* KO tumours compared with *Pten/Trp53/Kmt9α* KO or Ctrl prostates (Fig. 1I). These chemokines bind to and activate the CXCR2 receptor. The CXCL5/CXCR2 interaction is crucial for the recruitment and activation of neutrophils and promotes prostate tumour progression [31]. We validated the transcriptome data by qRT-PCR (Fig. 1J). Furthermore, using a proteome profiler array, we confirmed very low protein levels of CXCL1, CXCL2, and CXCL5 in Ctrl mice. In contrast, these chemokines were present at high levels in *Pten/Trp53* KO prostates but strongly decreased upon additional deletion of *Kmt9α* (Fig. 1K and Supplemental Fig. 1M-O). Together, our findings support the idea that KMT9 accounts for the expression of chemokines responsible for the recruitment of immunosuppressive PMN-MDSCs in *Pten/Trp53* KO prostate tumours.

To further validate that these chemokines were expressed in a KMT9-dependent manner in *Pten/Trp53* KO prostate tumour cells, we performed single-cell (sc)RNA-seq with Ctrl, *Pten/Trp53* KO, and *Pten/Trp53/Kmt9α* KO prostates. Unsupervised clustering and two-dimensional embedding using uniform manifold approximation and projection (UMAP) of Ctrl, *Pten/Trp53* KO, and *Pten/Trp53/Kmt9α* KO scRNA-seq data revealed nine cell sub-populations based on the expression of established marker genes (Supplemental Fig. 1P). Among these sub-populations, we identified “epithelial luminal” and “immune” cell populations (Supplemental Fig. 1Q, R). Population-dependent gene expression analyses showed that prostate epithelial luminal cells expressed the highest level of *Kmt9α* mRNA of all cell populations identified (Supplemental Fig. 1S). A marked decrease in *Kmt9α* mRNA levels in the epithelial luminal cell population was observed, as expected, in *Pten/Trp53/Kmt9α* KO compared with *Pten/Trp53* KO prostates (Supplemental Fig. 1T). In both, *Pten/Trp53* KO and *Pten/Trp53/Kmt9α* KO luminal cells, we also observed a decrease in *Pten* and *Trp53* levels compared with cells from Ctrl prostates (Supplemental Fig. 1U,V). Analysis of the expression of *Cxcl* chemokines revealed that *Pten/Trp53* KO luminal cells expressed high levels of *Cxcl 2, 3, 5* (Fig. 1L). Importantly, in the absence of KMT9α, expression of these chemokines in luminal cells was reduced providing further evidence for KMT9-controlled expression.

Next, we analysed by ChIP-seq the genomic localisation of KMT9α in *Pten/Trp53* KO prostate luminal cells established from our mouse model (Supplemental Fig. 1W). Analysis of the ChIP-seq data revealed 7,888 locations occupied by KMT9α at gene promoters and indicated a strong KMT9α enrichment at both, the *Cxcl2* and the *Cxcl5* promoter (Fig. 1M and Supplemental Fig. 1X). Promoter occupation by KMT9α correlated with *Cxl2* and *Cxcl5* mRNA expression, which was higher in *Pten/Trp53* KO than in Ctrl cells (Fig. 1N), lower in *Pten/Trp53/Kmt9α* KO than in *Pten/Trp53* KO cells, and restored upon transduction of cells with a nuclear localisation signal (NLS)-tagged KMT9α (*Pten/Trp53/Kmt9α* KO/NLS-KMT9α cells) (Fig. 1N and Supplemental Fig. 1Y). Using a proteome profiler array, we confirmed that distinct mRNA levels correlated with protein expression (Fig. 1O and Supplemental Fig. 1Z, AA). Together, these findings indicate that in *Pten/Trp53* KO prostate tumour epithelial cells, KMT9 controls expression of chemokines that are associated with recruitment of PMN-MDSCs and the formation of an immunosuppressive TIME.

### Kmt9α ablation impairs PMN-MDSC recruitment and favours T cell infiltration of the prostate TIME

Intra-tumoural PMN-MDSC infiltration has been identified as a major cause of immunosuppression and PCa progression [32]. Accordingly, increased levels of immature myeloid precursors and neutrophils have been observed in the blood of patients with advanced stages of PCa [13, 33]. Thus, we next analysed the levels of these immune cell populations in the blood of our mouse models. Consistent with observations in human prostate tumour patients [13], we found a significant increase in circulating myeloid (CD11b^+^) and neutrophils (CD11b^+^ Ly6G^+^) in the blood of *Pten/Trp53* KO compared with Ctrl mice (Supplemental Fig. 2A-D). In contrast, the number of myeloid cells and neutrophils in the blood of *Pten/Trp53/Kmt9α*KO mice was reduced to about Ctrl levels (Supplemental Fig. 2A-D).

**Fig. 2:**
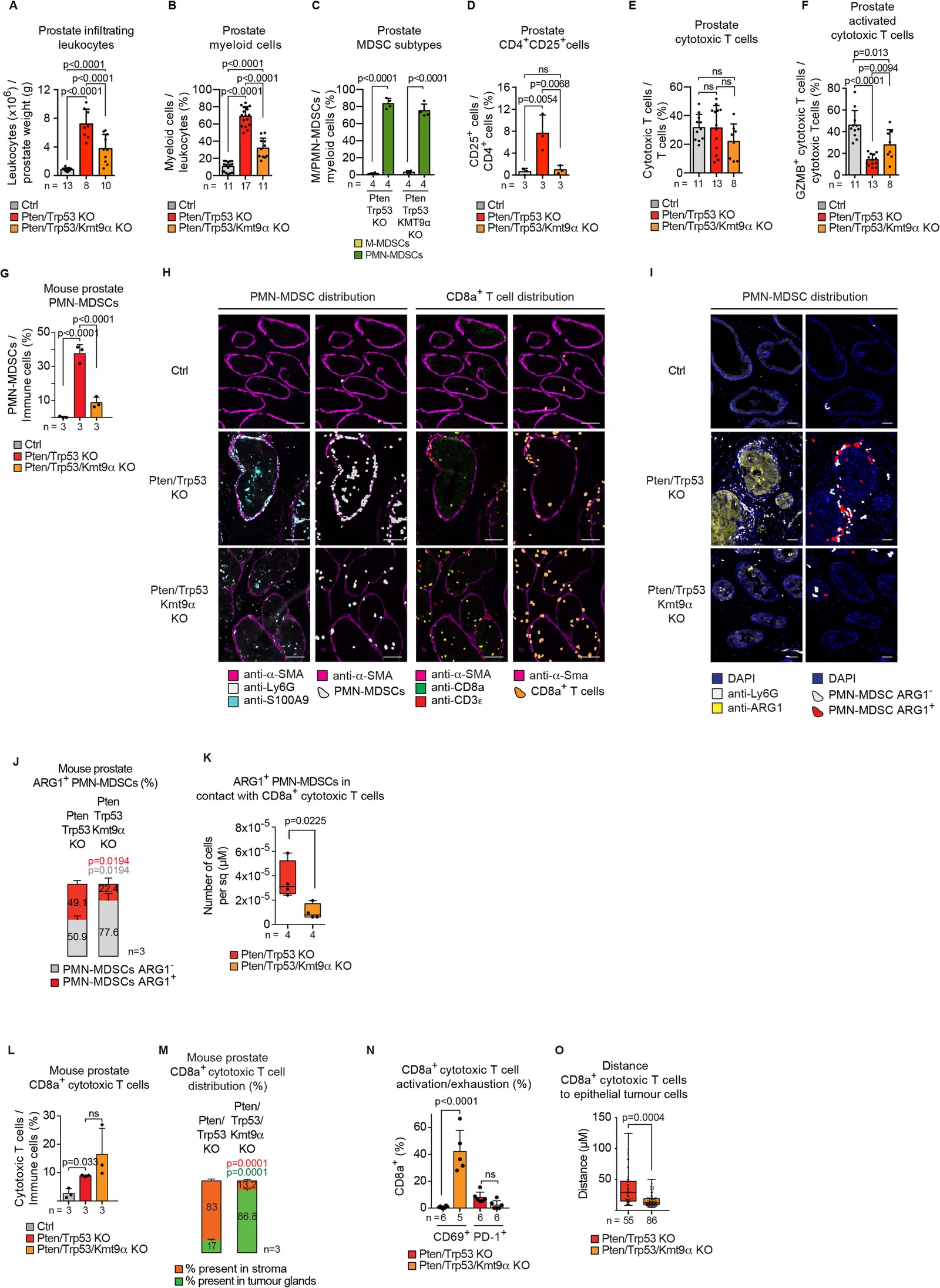
*Kmt9α* loss blocks infiltration of PMN-MDSCs and favours presence of activated cytotoxic T cells in *Pten/Trp53/Kmt9α* KO prostate tumour glands. **A**-**F**, Multiparametric flow cytometry analysis of tumour-infiltrating leukocytes isolated from Ctrl, *Pten/Trp53* KO, and *Pten/Trp53/Kmt9α* KO mice prostates. **A**, Relative numbers of prostate-infiltrating leukocytes (CD45^+^) found in Ctrl, *Pten/Trp53* KO, and *Pten/Trp53/Kmt9α* KO mice prostates. **B**, Percentages of myeloid cells (CD45^+^ CD11b^+^) found in prostates. **C**, Subtyping of prostate infiltrating MDSCs into M-MDSCs (CD45^+^ CD11b^+^ Ly6G^−^ Ly6C^hi^) and PMN-MDSCs (CD45^+^ CD11b^+^ Ly6G^+^ Ly6C^lo^). **D**-**F**, Analysis of lymphocyte infiltration and activation in Ctrl, *Pten/Trp53* KO, and *Pten/Trp53/Kmt9α* KO mice prostates. Percentage of infiltrating CD45^+^ CD3^+^ CD4^+^ CD25^+^ cells (**D**), cytotoxic T cells (CD45^+^ CD3^+^ CD8a^+^) or (**E**), activated cytotoxic T cells (CD45^+^, CD3^+^, CD8a^+^, GZMB^+^) (**F**) found in prostates. n, represents the number of prostates analysed. **G**, Bar graphs representing distribution and percentages of PMN-MDSCs in Ctrl, *Pten/Trp53* KO, and *Pten/Trp53/Kmt9α* KO prostates identified by single-cell spatial phenotyping (*n* = 3 prostates). **H**, PMN-MDSC and cytotoxic T cell distribution in Ctrl, *Pten/Trp53* KO, and *Pten/Trp53/Kmt9α* KO prostates unravelled by single-cell phenotyping. Cell types and antibodies used for staining are indicated. Scale bars represent 100 µm. **I, J**, Representative images (**I**) and quantification (**J**) of ARG1-expressing PMN-MDSCs in Ctrl, *Pten/Trp53* KO and *Pten/Trp53/Kmt9α* KO prostates (*n* = 3). Scale bars represent 100 µm. **K**, Box-Whisker plots with min and max values representing Ly6G^+^ ARG1^+^ PMN-MDSCs in contact with CD8a^+^ T cells in *Pten/Trp53* KO and *Pten/Trp53/Kmt9α* KO prostates (*n* = 4 prostate samples). **L,** Bar graphs representing distribution and percentages of cytotoxic T cells in Ctrl, *Pten/Trp53* KO, and *Pten/Trp53/Kmt9α* KO prostates identified by single-cell spatial phenotyping (*n* = 3 prostate samples). **M**, Bar graph representing distribution of cytotoxic T cell in *Pten/Trp53* KO and *Pten/Trp53/Kmt9α* KO prostates (*n* = 3 prostate samples). **N**, Bar graph representing percentage of activated (CD69^+^) and exhausted (PD-1^+^) CD8a^+^ T cells in *Pten/Trp53* KO and *Pten/Trp53/Kmt9α* KO prostates. n, represent the number of prostate samples analysed. **O**, Box-Whisker plots with min and max values representing the distance of cytotoxic T cells to epithelial tumour cells in *Pten/Trp53* KO and *Pten/Trp53/Kmt9α* KO prostates. Data represent means + s.d. (**A**-**G**, **J**, **L**-**N**). *P* values obtained by unpaired Student T test (**O**), one-way ANOVA test (**A**-**G**, **L**, **N**) or two-way ANOVA test (**J**, **K**, **M**) are indicated

To address the question whether differences in neutrophil and myeloid numbers observed in the blood of *Pten/Trp53* KO and *Pten/Trp53/Kmt9α* KO mice correlated with distinct TIMEs, we isolated and quantified CD45^+^ infiltrating leukocytes from mouse prostates. We found a massive infiltration of leukocytes in *Pten/Trp53* KO tumours that was not observed in Ctrl and significantly reduced in *Pten/Trp53/Kmt9α* KO prostates (Fig. 2A). Multi-parametric flow cytometry analyses revealed that most of the tumour-infiltrating leukocytes in *Pten/Trp53* KO tumours were myeloid cells (CD11b^+^) (Fig. 2B and Supplemental Fig. 2E). MDSCs can be classified as monocytic (M)-MDSCs (CD11b^+^ Ly6G^−^ Ly6C^hi^) or PMN-MDSCs (CD11b^+^ Ly6G^+^ Ly6C^lo^) [18]. Since PMN-MDSCs have similar morphologic and phenotypic characteristics as neutrophils [34], we confirmed by flow cytometry analysis that most of the infiltrating-myeloid cells present in both *Kmt9α* proficient and deficient tumours were PMN-MDSCs (Fig. 2C and Supplemental Fig. 2F). Furthermore, in *Pten/Trp53/Kmt9α* KO prostate tumours, the infiltration of CD4^+^ CD25^+^ cells was suppressed (Fig. 2D and Supplemental Fig. 2G). Although *Kmt9α* loss did not affect infiltration of cytotoxic T cells (CD3^+^ CD8a^+^) (Fig. 2E and Supplemental Fig. 2H), it increased cytotoxic T cell activity (CD3^+^ CD8a^+^ GZMB^+^) in *Pten/Trp53/Kmt9α* KO tumours (Fig. 2F and Supplemental Fig. 2I).

Next, we performed single-cell spatial phenotyping in mouse prostates using a panel of 28 antibodies targeting immune markers (CD45, CD44, CD3ε, CD20, CD68, CD11c, CD31, CD8a, CD45R/B220, FOXP3, CD36, CD86, FcRy, TER119), myeloid markers (F4/80, Ly6G, IBA1, CD206, S100A9), structural markers (VIM, COL1A1, CAV1, α-SMA, CTNNB1), proliferation markers (KI67, PCNA), and PD-L1. We also included a pan-keratin (KRT) antibody, since expression analyses revealed that *Kmt9α* ablation strongly reduces expression of epithelial cell markers such as *Krt4*, *−5*, *−6a*, *−7*, *−8*, *−14*, *−15*, *−16,* and *−19* (Supplemental Fig. 2J). The pan-KRT antibody allows for selective identification of KMT9α proficient and deficient epithelial cells in *Pten/Trp53* KO and *Pten/Trp53/Kmt9α* KO prostate tissue.

Single-cell spatial phenotyping detected normal and tumour epithelial cells, proliferating cells, fibroblasts, and immune cell populations (Supplemental Fig. 2K-M). In total, single-cell phenotyping allowed us to identify 11 distinct cell phenotypes across Ctrl, *Pten/Trp53* KO, and *Pten/Trp53/Kmt9α* KO prostates (Supplemental Fig. 2L, M). Normal prostate epithelial cells were only present in Ctrl prostates (Supplemental Fig. 2N, O). In *Pten/Trp53* KO and *Pten/Trp53/Kmt9α* KO prostates, however, we observed the appearance of distinct populations of tumour epithelial cells. A population expressing KRT levels (KRT^high^) was mainly observed in *Pten/Trp53* KO prostates. In contrast, in *Pten/Trp53/Kmt9α* KO prostates, we found a population expressing low levels of KRT (KRT^low^) (Supplemental Fig. 2L-O). These data are in accordance with our RNA-seq and qRT-PCR analyses (Fig. 1G and Supplemental Fig. 2J). Upon *Kmt9α* ablation we observed reduced numbers of proliferating cells (Supplemental Fig. 2N-O). Single-cell spatial phenotyping further revealed the presence of PMN-MDSCs (Ly6G^+^ S100A9^+^) [35] representing the vast majority of immune cells (Fig. 2G) infiltrating *Pten/Trp53* KO prostate tumours. PMN-MDSCs formed a barrier surrounding the prostate tumour glands (Fig. 2H). Among the PMN-MDSCs, about 50% had an immunosuppressive phenotype (Ly6G^+^ ARG1^+^, Fig. 2I, J) and were in contact with cytotoxic T cells (CD8a^+^, Fig. 2K) representing about 10% of immune cells infiltrated in *Pten/Trp53* KO prostate tumours (Fig. 2L). Most of the cytotoxic T cells (83%) were located in the stroma (Fig. 2H, M) and exhausted (PD-1^+^) while only few were activated (CD69^+^, 1%) (Fig. 2N). Strikingly, in *Pten/Trp53/Kmt9α* KO tumour prostate glands, our analysis uncovered a reduced percentage of PMN-MDSCs (Fig. 2G) as well as immunosuppressive ARG1^+^ PMN-MDSCs (Fig. 2J) and the dismantling of the PMN-MDSC barrier (Fig. 2H, I). As a consequence, most of the cytotoxic T cells (87%) were relocated into the tumour glands (Fig. 2H, M) with reduced distances to epithelial tumour cells (Fig. 2O). On the other hand, fewer cytotoxic T cells were found in contact with immunosuppressive ARG1^+^ PMN-MDSCs (Fig. 2K). Accordingly, we observed increased percentages of activated (CD69^+^) and decreased percentages of exhausted (PD-1^+^) CD8a^+^ cytotoxic T cells (Fig. 2N). Together, these data show that KMT9 controls the infiltration and the spatial distribution of PMN-MDSCs, which accounts for the exclusion of cytotoxic T cells from *Pten/Trp53* KO prostate tumours glands.

### Kmt9α ablation combined with CXCR2 inhibition further reduces prostate tumour growth

**Fig. 3:**
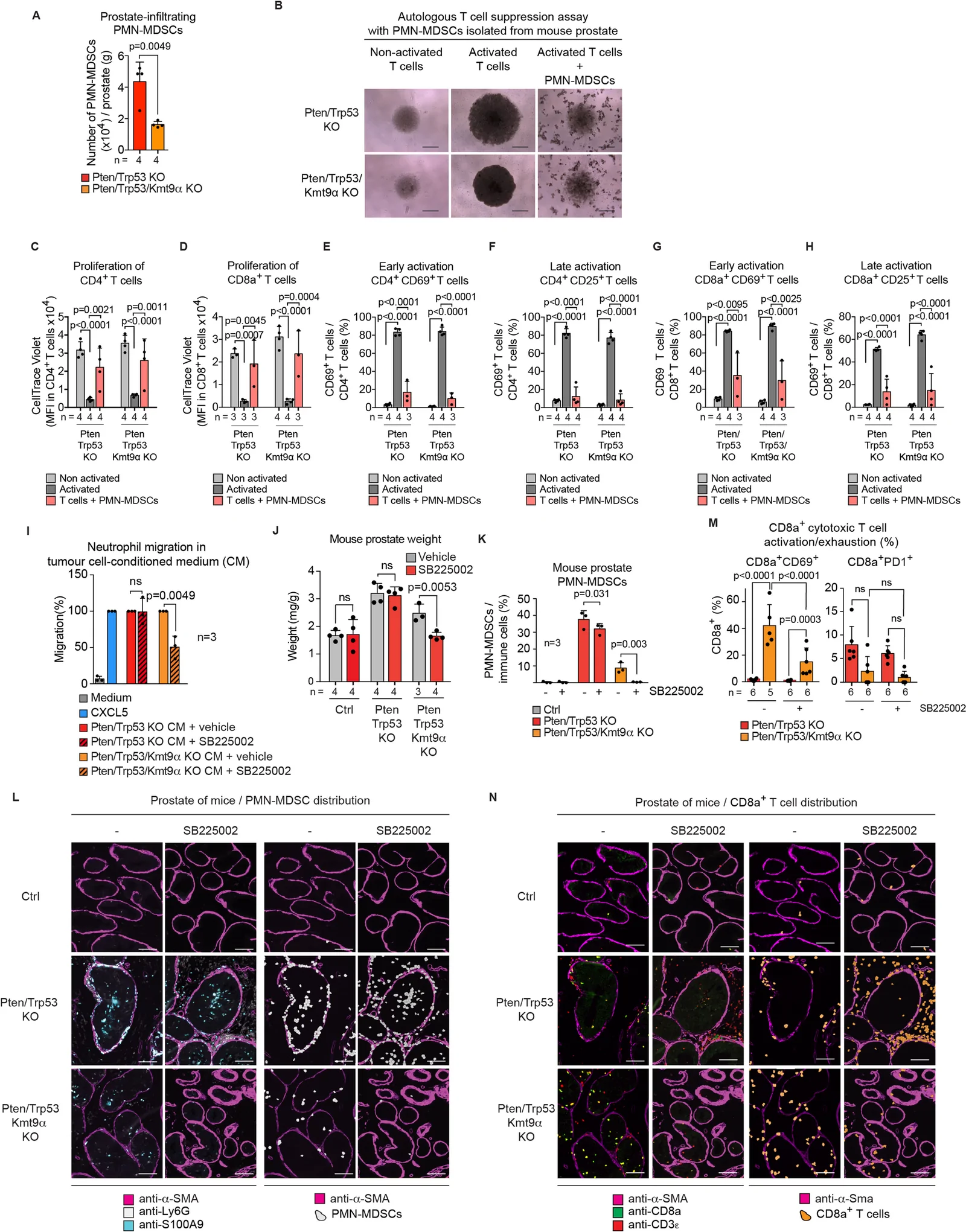
*Kmt9α* ablation combined with CXCR2 inhibition results in increased inhibition of prostate tumour growth. **A**, Relative numbers of prostate-infiltrating PMN-MDSCs found in Ctrl, *Pten/Trp53* KO, and *Pten/Trp53/Kmt9α* KO mice prostates (n = 4). n, represents the number of prostates analysed. **B**, Representatives pictures of non-activated T cells, T cells activated with anti-CD3/CD28 beads and activated T cells co-cultured with PMN-MDSCs isolated from either *Pten/Trp53* KO or *Pten/Trp53/Kmt9α* KO prostates. **C**, **D**, Relative CD4^+^ and CD8a^+^ T cell proliferation evaluated by dilution of CellTRace Violet in non-activated T cells, in T cells activated with anti-CD3/CD28 beads and in activated T cells cocultured with PMN-MDSCs isolated from either *Pten/Trp53* KO or *Pten/Trp53/Kmt9a* KO prostate. (*n* = 3) **E**–**H**, Percentage of CD69^+^ CD4^+^ (**E**), CD25^+^ CD4^+^(**F**), CD69^+^ CD8a^+^ (**G**), CD25^+^ CD8a^+^ (**H**) T cells when non-activated, when activated with anti-CD3/CD28 beads and when activated T cells were cocultured with PMN-MDSCs isolated from either *Pten/Trp53* KO or *Pten/Trp53/Kmt9α* KO prostates. n, represent the number of biological replicates. **I**, Migration of neutrophil isolated from bone marrow was assessed in transwell chemotaxis assays in medium (Advanced DMEM/F12), in medium supplemented with 100 ng/ml mouse CXCL5, in *Pten/Trp53* KO or in *Pten/Trp53/Kmt9α* KO conditioned media (CM) as indicated. Neutrophils were either vehicle-treated or treated with the CXCR2 inhibitor SB225002. n, represent the number of biological replicates. **J**, Bar graph representing the relative weight of prostates isolated from Ctrl, *Pten/Trp53* KO, and *Pten/Trp53/Kmt9α* KO mice treated with vehicle (*n* = 4 mice for Ctrl and *Pten/Trp53* KO; *n* = 3 mice for *Pten/Trp53/Kmt9α* KO) or SB225002 (*n* = 4 mice for Ctrl, *Pten/Trp53* KO, and *Pten/Trp53/Kmt9α* KO). **K**, Bar graphs representing percentages of PMN-MDSCs in Ctrl, *Pten/Trp53* KO, and *Pten/Trp53/Kmt9α* KO prostates of mice treated with either vehicle or with SB225002 and identified by single-cell spatial phenotyping (*n* = 3). n, represents the number of prostate samples analysed. **L**, PMN-MDSC distribution in Ctrl, *Pten/Trp53* KO, and *Pten/Trp53/Kmt9α* KO prostates of mice treated with vehicle or SB225002 unravelled by single-cell phenotyping. Cell types and antibodies are indicated. **M**, Bar graphs representing percentages of activated CD69^+^ CD8a^+^ and exhausted PD-1^+^ CD8a^+^ T cells in Ctrl, *Pten/Trp53* KO, and *Pten/Trp53/Kmt9α* KO prostates of mice treated with vehicle or SB225002 identified by single-cell spatial phenotyping. n, represents the number of prostate samples analysed. **N**, CD8a^+^ cytotoxic T cells distribution in Ctrl, *Pten/Trp53* KO, and *Pten/Trp53/Kmt9α* KO prostates of mice treated with vehicle or SB225002 unravelled by single-cell phenotyping. Cell types and antibodies are indicated. Scale bars represent 500 µm (**B**) and 100 µm (**L**, **N**). Data represent means + s.d. (**A**, **C**-**K**, **M**). P values obtained by unpaired Student T test (**A**) and one-way ANOVA test (**C-K, M**) are indicated

Next, to functionally characterize their immunosuppressive properties in *Pten/Trp53* KO and *Pten/Trp53/Kmt9α* KO prostate tumours, infiltrating PMN-MDSCs were isolated and cocultured with autologous T cells that were activated with anti-CD3/CD28 beads. Subsequently, T cell activity and proliferation were assessed. Consistent with previous observations (Fig. 2B, C, G-I), we found a significant decrease in the total number of prostate-infiltrating PMN-MDSCs in *Pten/Trp53/Kmt9α*  KO compared to *Pten/Trp53* KO prostates (Fig. 3A). Importantly, we observed that both prostate-infiltrating PMN-MDSC populations blocked T cell proliferation (Fig. 3B). We confirmed by flow cytometry that both PMN-MDSC populations impaired CD4^+^ and CD8a^+^ T cells proliferation (Fig. 3C, D). Additionally, in the presence of PMN-MDSCs, we observed decreased percentages of CD69^+^ (early activation) and CD25^+^ (late activation) populations of CD4^+^ and CD8a^+^ T cells (Fig. 3 E–H). These data show that prostate infiltrating PMN-MDSCs suppress T cell proliferation and activity, demonstrating their immunosuppressive potential. Notably, our findings indicate that KMT9α loss in *Pten/Trp53/Kmt9α*  KO prostate tumours does not alter the immunosuppressive properties of PMN-MDSCs.

While our data show that *Kmt9α* deficiency reduces the expression of most myeloid chemoattracting CXCR2 ligands (Fig. 1I-L, N), it does not completely block the expression of CXCL5 chemokines (Fig. 1K). Since CXCR2^+^ PMN-MDSCs remain present in *Pten/Trp53/Kmt9α* KO prostates (Supplemental Fig. 3A, B), we hypothesized that CXCR2 inhibition in these mice might further increase the anti-tumoural response. To validate our idea, we first evaluated the migratory capacity of vehicle-treated neutrophils and neutrophils treated with the CXCR2 inhibitor SB225002 in response to chemotactic gradients generated with *Pten/Trp53* KO and *Pten/Trp53/Kmt9α*  KO conditioned medium (CM). While CXCR2 inhibition failed to block neutrophil migration in the chemotactic gradient generated with *Pten/Trp53* KO CM, migration of SB225002-treated neutrophil was found significantly decreased in the chemotactic gradient generated with *Pten/Trp53/Kmt9α*  KO CM compared to vehicle-treated neutrophils (Fig. 3I). These findings support our hypothesis that the reduced chemokine production resulting from KMT9 depletion in *Pten/Trp53/Kmt9α*  KO cells increases the sensitivity of neutrophil migration to CXCR2 inhibition. In *Pten/Trp53* KO cells, the KMT9-dependent production of chemokines might not be overcome by SB225002 that acts as a competitive antagonist, thereby reducing treatment efficacy. To further validate this hypothesis, *Pten/Trp53* KO, *Pten/Trp53/Kmt9α* KO, and Ctrl mice were then treated daily for fifteen days with SB225002, a potent competitive CXCR2 inhibitor, and then sacrificed for analysis (Supplemental Fig. 3C). Treatment with SB225002 neither affected the overall weight of the mice (Supplemental Fig. 3D) nor the weight of Ctrl or *Pten/Trp53* KO prostates (Fig. 3J). However, whereas *Pten/Trp53* KO mice did not respond to SB225002, *Pten/Trp53/Kmt9α* KO treated-mice showed a significant decrease in prostate weight compared with vehicle-treated mice, suggesting treatment-induced tumour regression (Fig. 3J).

To further investigate the combined effect of CXCR2 inhibition and *Kmt9α* loss, we performed single-cell spatial phenotyping. Staining with anti-SMA and anti-Pan-KRT antibodies showed that treatment with SB225002 did not affect the overall gland structure in Ctrl, *Pten/Trp53* KO and *Pten/Trp53/Kmt9α* KO prostates (Supplemental Fig. 3E). Furthermore, SB225002 had a minor impact on PMN-MDSC infiltration in Ctrl and *Pten/Trp53* KO prostates as revealed by staining with anti-Ly6G and anti-S100A9 antibodies (Fig. 3K, L). In sharp contrast, SB225002 treatment abrogated PMN-MDSC infiltration of tumour glands in *Pten/Trp53/Kmt9α* KO prostates (Fig. 3K, L). In line with these results, we observed fewer exhausted (PD1^+^) and more activated (CD69^+^) CD8^+^ cytotoxic T cells in *Pten/Trp53/Kmt9α* KO prostates than in *Pten/Trp53* KO prostates of mice treated with SB225002 (Fig. 3M). Importantly, despite finding no significant differences in CD8^+^ T cell infiltration in response to SB225002 treatment in both *Pten/Trp53* KO and *Pten/Trp53/Kmt9α* KO prostates (Supplemental Fig. 3F), CD8a^+^ T cells were present in tumour glands of SB225002-treated *Pten/Trp53/Kmt9α* KO mice but rarely observed in glands of SB225002-treated *Pten/Trp53* KO mice due to the presence of PMN-MDSCs barriers around glands (Fig. 3N). Together, these data indicate that inhibition of CXCR2 combined with *Kmt9α* ablation elicits a more pronounced reduction of prostate tumour growth than *Kmt9α* loss alone.

### KMT9-induced ARG1 expression impairs T cell cytotoxicity in prostate cancer

**Fig. 4:**
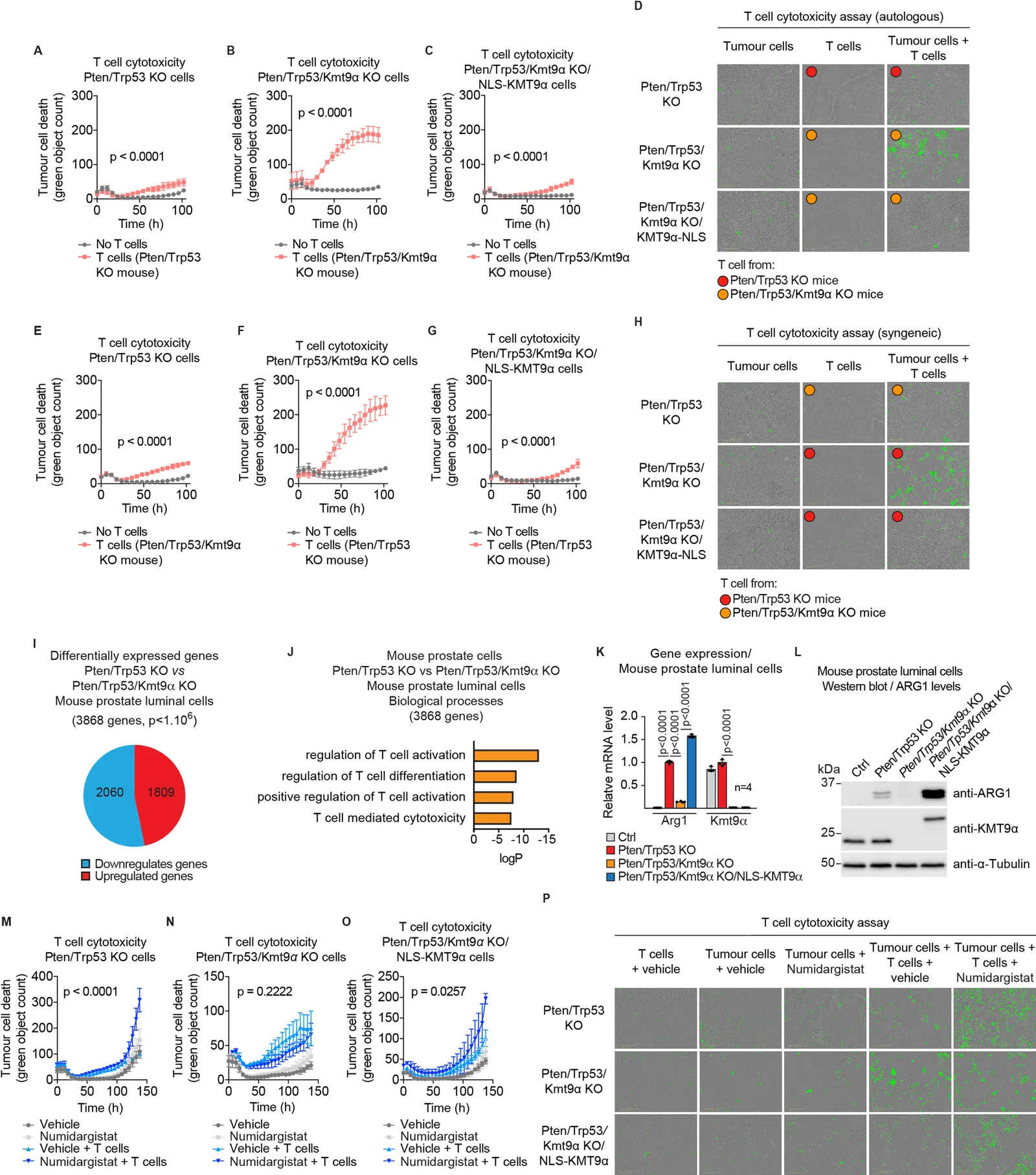
KMT9-regulated expression of ARG1 impairs T cell cytotoxicity in PCa. **A**-**H**, Autologous and syngeneic T cell-mediated cytotoxicity against prostate tumour cells was determined by measuring tumour cell death. Autologous (**A**-**D**) or syngeneic (**E**–**H**) T cells activated with anti-CD3/CD28 beads were cocultured with either *Pten/Trp53* KO, *Pten/Trp53/Kmt9α* KO or *Pten/Trp53/Kmt9α* KO/NLS-KMT9α (*n* = 3). n, represents the number of biological replicates. Representative images of autologous (**D**) or allogenic (**H**) T cell-mediated cytotoxicity against prostate tumour cells. **I**, Diagram showing the number of genes differentially expressed between *Pten/Trp53* KO and *Pten/Trp53/Kmt9α* KO mouse prostate luminal cells. **J**, Enriched pathway analysis for 3868 DEGs between *Pten/Trp53* KO and *Pten/Trp53/Kmt9α* KO prostate tumour cells. Bars represent individual Benjamini logP values derived from GO enrichment analysis. **K**, QRT-PCR analysis showing relative mRNA levels of *Arg1* and *Kmt9α* (n = 4 biological independent replicates) in Ctrl, *Pten/Trp53* KO, *Pten/Trp53/Kmt9α* KO and *Pten/Trp53/Kmt9α* KO/NLS-KMT9α prostate luminal cells. Data represent means + s.d. **L**, Analysis of ARG1 protein expression by Western blot of Ctrl, *Pten/Trp53* KO, *Pten/Trp53/Kmt9α* KO and *Pten/Trp53/Kmt9α* KO/NLS-KMT9α prostate cells. α-Tubulin served as control. **M**–**O**, Autologous T cell-mediated cytotoxicity against prostate tumour cells in presence or absence of the ARG1 inhibitor numidargistat was determined by measuring tumour cell death. Autologous T cells activated with anti-CD3/CD28 beads were cocultured with *Pten/Trp53* KO (**M**), *Pten/Trp53/Kmt9α* KO (**n**), and *Pten/Trp53/Kmt9α* KO/NLS-KMT9α (**O**) prostate tumour cells in presence of either vehicle or numidargistat as indicated. **P**, Representative images of autologous T cell-mediated cytotoxicity against prostate tumour cells cultured in presence of either vehicle or numidargistat (n = 3 biological independent replicates). Scale bars represent 400 µm (**D**, **H**, **P**). Data represent means ± s.d. (**A**-**C**, **E**–**G**, **K**, **M**–**O**). P values obtained by unpaired Student T test (**K**) and by two-way ANOVA test (**A**-**C**, **E**–**G**, **M**–**O**) are indicated

Our findings show that upon *Kmt9α*  loss in *Pten/Trp53/Kmt9α*  KO prostate the immunosuppressive PMN-MDSC barrier surrounding prostate tumour glands is dismantled and CD8a^+^ cytotoxic T cells are found activated and relocated into the tumour glands where they are in close contact with epithelial tumour cells (Fig. 2H). Thus, we wondered whether *Pten/Trp53/Kmt9α*  KO prostate tumour cells are sensitive to T cell-mediated cytotoxicity. Accordingly, we performed T cell cytotoxicity assays by coculturing *Pten/Trp53* KO, *Pten/Trp53/Kmt9α*  KO or *Pten/Trp53/Kmt9α*  KO/NLS-KMT9α cells with autologous T cells that were isolated from *Pten/Trp53* KO or *Pten/Trp53/Kmt9α*  KO mice spleens and activated with anti-CD3/CD28 beads. We uncovered that T cells from *Pten/Trp53* KO mice had a mild effect on *Pten/Trp53* KO cell viability (Fig. 4A, d and Supplemental Fig. 4A). In comparison, T cells from *Pten/Trp53/Kmt9α*  KO mice displayed a remarkable cytotoxic and anti-proliferative effect on *Pten/Trp53/Kmt9α*  KO cells (Fig. 4B, D, and Supplemental Fig. 4B). Of note, ectopic expression of NLS-KMT9α in *Pten/Trp53/Kmt9α*  KO/NLS-KMT9α restored resistance toward T cell-mediated cytotoxicity (Fig. 4C, D and Supplemental Fig. 4C). Previous research demonstrated that cytotoxic activity of splenic T cells is affected in tumour-bearing mice [36]. Thus, we aimed to explore whether the differences found in T cell cytotoxicity are due to differences in splenic T cell status. To that end, *Pten/Trp53* KO cells were cocultured with T cells isolated from *Pten/Trp53/Kmt9α*  KO mice spleen and *Pten/Trp53/Kmt9α*  KO and *Pten/Trp53/Kmt9α*  KO/NLS-KMT9α cells were cocultured with T cells isolated from *Pten/Trp53* KO mice spleen. Strikingly, syngeneic T cells showed only a clear cytotoxic effect on *Pten/Trp53/Kmt9α*  KO cells (Fig. 4E-H and Supplemental Fig. 4D-F). Together, these findings demonstrate that loss of *Kmt9α* in cells sensitizes prostate tumour cells to T cell-mediated cytotoxicity.

To discover the KMT9-dependent mechanisms regulating resistance of *Pten/Trp53 KO* cells to T cell-mediated cytotoxicity, we compared the transcriptomes of *Pten/Trp53* KO and *Pten/Trp53/Kmt9α*  KO cells and found 3,868 DEGs (Fig. 4I). Gene enrichment analyses for these DEGs uncovered biological processes including regulation of T cell activation and differentiation, and T cell-mediated cytotoxicity among the top-ranking categories (Fig. 4J). Among the DEGs, we found that *Arg1* mRNA expression levels were increased in *Pten/Trp53 KO* cells compared to Ctrl cells (Fig. 4K). In *Pten/Trp53/Kmt9α*  KO cells, we observed decreased expression of *Arg1* that was restored in *Pten/Trp53/Kmt9α*  *KO*/NLS-KMT9α cells (Fig. 4K). Furthermore, western blot analysis confirmed that ARG1 protein expression is absent in Ctrl cells but induced in *Pten/Trp53* KO cells. This expression decreased in *Pten/Trp53/Kmt9α* KO cells and was highly upregulated upon NLS-KMT9α expression in *Pten/Trp53/Kmt9α*/NLS-KMT9α cells, demonstrating that KMT9 regulates ARG1 expression (Fig. 4L). To investigate the molecular mechanism by which KMT9 regulates *Arg1* expression, we performed ChIP-seq for KMT9α. Our ChIP-seq datasets revealed no direct binding or enrichment of KMT9α at the proximal promoter region of *ARG1* (Supplemental Fig. 4G), suggesting that KMT9 does not act as a direct transcriptional activator at the *ARG1* locus. Together, these data suggest that KMT9 regulates *Arg1* expression via an indirect transcriptional mechanism.

Since tumour cells have been shown to mediate immunosuppression via ARG1 [14], we hypothesized that inhibition of ARG1 in *Pten/Trp53* KO prostate tumour cells might sensitize them to T cell-mediated cytotoxicity. Thus, we cocultured *Pten/Trp53* KO cells with T cells in presence or absence of the ARG1 inhibitor numidargistat. Importantly, as expected for an immunomodulatory mechanism that acts by restoring T-cell function rather than directly inducing tumour cell death, the effect of ARG1 inhibition developed progressively over the course of the co-culture. At the end of the experiment, numidargistat treatment resulted in an approximately threefold increase in T-cell-mediated cytotoxicity compared with vehicle-treated controls in *Pten/Trp53* KO cells (Fig. 4M, P). In *Pten/Trp53/Kmt9α*  KO cells, where ARG1 expression is downregulated, treatment with numidargistat had no effect (Fig. 4N, P). In contrast, the KMT9-dependent high levels of ARG1 in *Pten/Trp53/Kmt9α*  KO/NLS-KMT9α cells rendered cells sensitive to T cell-mediated cytotoxicity upon treatment with numidargistat (Fig. 4O, P). Together, our findings unravel that KMT9-regulated expression of ARG1 in tumour cells confers tumour cell resistance to T cell-mediated cytotoxicity.

### Kmt9α loss combined with ARG1 inhibition results in increased inhibition of prostate tumour growth

**Fig. 5:**
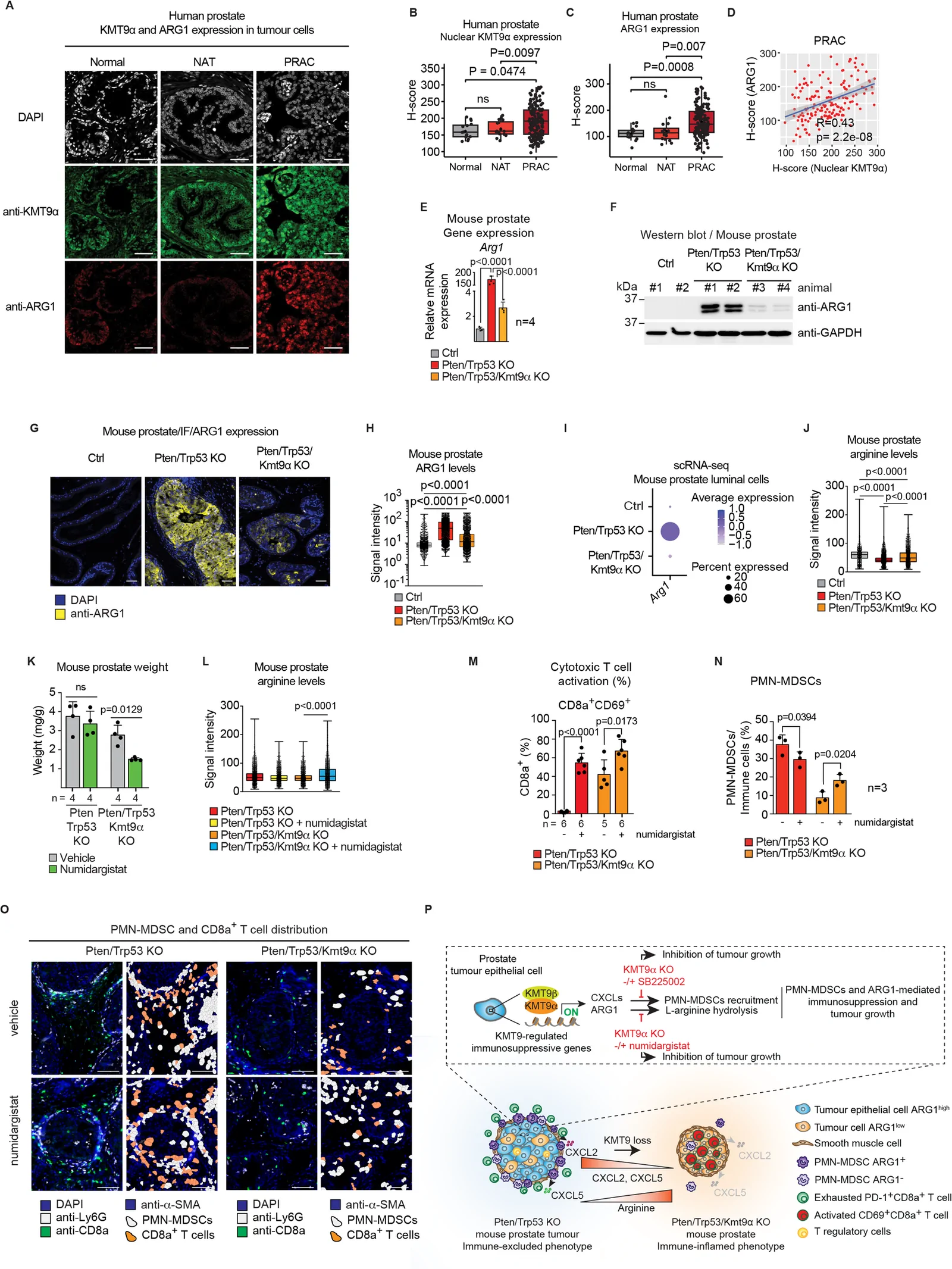
K*mt9α* loss combined with ARG1 inhibition results in increased inhibition of prostate tumour growth. **A**-**C**, Representative images (**A**) and box-Whisker plots with min to max values showing quantification of KMT9α (**B**) or ARG1 (**C**) expression in human PCa. **D**, Correlation analysis of KMT9α and ARG1 expression in human PCa. **E**, QRT-PCR analysis showing relative mRNA levels of *Arg1* (n = 4) in Ctrl, *Pten/Trp53* KO, and *Pten/Trp53/Kmt9α* KO prostates. Data represent means + s.d. **F**, Analysis of ARG1 protein expression by Western blot of Ctrl, *Pten/Trp53* KO, and *Pten/Trp53/Kmt9α* KO prostates. **G**, **H**, Distribution (**G**) and Box-Whisker plots with min to max values showing signal intensity quantification (**H**) of ARG1 expression of Ctrl, *Pten/Trp53* KO, and *Pten/Trp53/Kmt9α* KO prostates. **I**, Dot blot displaying single-cell mRNA expression analysis of *Arg1* in luminal cells of Ctrl, *Pten/Trp53* KO, and *Pten/Trp53/Kmt9α* KO prostates. **J**, Box-Whisker plots with min to max values showing quantification of arginine levels of Ctrl, *Pten/Trp53* KO, and *Pten/Trp53/Kmt9α* KO prostates. **K**, Bar graph representing the relative weight of prostates isolated from *Pten/Trp53* KO and *Pten/Trp53/Kmt9α* KO mice treated with vehicle or numidargistat. **L**, Box-Whisker plots with min to max values showing quantification of arginine levels of *Pten/Trp53* KO and *Pten/Trp53/Kmt9α* KO prostates treated with vehicle or numidargistat. **M**, Bar graphs representing percentages of activated CD69^+^ CD8a^+^ T cells in *Pten/Trp53* KO and *Pten/Trp53/Kmt9α* KO prostates of mice treated with vehicle or numidargistat identified by single-cell spatial phenotyping using the Opal multiplex immunofluorescence staining system from Akoya. n, represents the number of prostate samples analysed. **N**, Bar graphs representing percentages of PMN-MDSCs in *Pten/Trp53* KO and *Pten/Trp53/Kmt9α* KO prostates of mice treated with either vehicle or with numidargistat and identified by single-cell spatial phenotyping using the PhenoCycler®-Fusion system from Akoya. n = 3 represents the number of prostate samples analysed. **O**, PMN-MDSC and CD8a^+^ cytotoxic T cell distribution in *Pten/Trp53* KO and *Pten/Trp53/Kmt9α* KO prostates of mice treated with vehicle or numidargistat unravelled by single-cell phenotyping. Cell types and antibodies are indicated. Scale bars represent 50 µm (**A**, **G**) and 100 µm (**O**). Data represent means + s.d. (**E**, **K**, **M**, **N**) or means. P values obtained by unpaired Student T test are indicated. n represents the number of biologically independent samples. **P**, In *Pten/Trp53* KO prostate tumour epithelial cells KMT9 regulates the expression of chemokines and ARG1 thereby promoting immunosuppression and tumour growth. Ablation of *Kmt9α* affects the composition and spatial organisation of immune cells in the TIME and impairs prostate tumour growth. Concomitant *Kmt9α* ablation and treatment with CXCR2 inhibitor (SB225002) or ARG1 inhibitor (numidargistat) results in increased inhibition of tumour growth

Increased levels of ARG1 have been observed in different cancer types including PCa, where the enzyme plays an immunosuppressive role by impairing T cell function [37]. Our analysis of human prostate tumour samples (PRAC, n = 160) revealed that the levels (H-scores) of nuclear KMT9α and ARG1 were significantly elevated in PCa tissue compared with normal (*n* = 16) and normal adjacent tissue (NAT, n = 16) (Fig. 5A-C). Furthermore, nuclear KMT9α and ARG1 levels correlated as depicted by H-score ratios (Fig. 5D). We also detected high ARG1 mRNA and protein levels in *Pten/Trp53* KO prostate tumour compared to Ctrl and *Pten/Trp53/Kmt9α* KO tumours (Fig. 5E-H). Importantly, loss of *Kmt9α* led to decreased levels of ARG1 in luminal cells of *Pten/Trp53/Kmt9α* KO prostates (Fig. 5G-I and Supplemental Fig. 5A). Consequently, we found increased levels of arginine in *Pten/Trp53/Kmt9α* KO compared with *Pten/Trp53* KO prostates (Fig. 5J). Since cytotoxic T cells are excluded from the tumour glands of *Pten/Trp53* KO prostates, we hypothesized that ARG1 inhibition with numidargistat would increase activation of cytotoxic T cells but without effect on tumour growth. In contrast, the treatment of *Pten/Trp53/Kmt9α* KO mice with numidargistat might enhance the anti-tumoural response given the presence of cytotoxic T cells in prostate tumour glands. Furthermore, while KMT9α ablation in *Pten/Trp53/Kmt9α* KO mice successfully suppresses ARG1 expression intrinsically within the prostate tumour cells, it does not eliminate ARG1 production from infiltrating PMN-MDSC ARG1^+^ cells which remain a potent source of stromal arginase (Fig. 2I,J). To neutralize this remaining, microenvironmental source of ARG1 that escapes genetic tumour-cell targeting, pharmacological inhibition with numidargistat is required. To validate our hypothesis, *Pten/Trp53* KO and *Pten/Trp53/Kmt9α* KO mice were treated daily with numidargistat for fifteen days and then sacrificed for analysis (Supplemental Fig. 5B). Treatment with numidargistat neither affected the overall weight of the mice (Supplemental Fig. 5C) nor tumour growth in *Pten/Trp53* KO mice (Fig. 5K). In contrast, in *Pten/Trp53/Kmt9α* KO mice treated with numidargistat, we observed increased arginine levels and a further decrease in prostate weight compared with vehicle-treated *Pten/Trp53/Kmt9α* KO mice (Fig. 5K, L).

To explain the combined effect of ARG1 inhibition and *Kmt9α *loss, we performed single-cell spatial phenotyping. While having no detectable effect on tumour growth in *Pten/Trp53* KO mice, numidargistat treatment caused a massive increase in activated CD69^+^ CD8a^+^ cytotoxic T cells (Fig. 5M). Despite an increased percentage of activated T cells, treatment with numidargistat had no effect on PMN-MDSC infiltration and barrier formation in *Pten/Trp53* KO tumours (Fig. 5N, O). CD8a^+^ cytotoxic T cells were found excluded from tumour glands of both vehicle and numidargistat-treated mice (Fig. 5O). In contrast, in Pten*/Trp53/Kmt9α* KO mice, we found a higher percentage of activated CD69^+^ CD8a^+^ cytotoxic T cells in numidargistat-treated mice compared to vehicle and presence of CD8a^+^ cytotoxic T cells in the tumour glands (Fig. 5M, O). Together, these data show a combined inhibitory effect of *Kmt9α* ablation and numidargistat treatment on prostate tumour growth.

In summary, we uncovered KMT9 as a major regulator of the immunosuppressive TIME in *Pten/Trp53* KO prostate tumours. KMT9 promotes tumour-infiltration of PMN-MDSCs, exclusion of cytotoxic T cell from tumour glands, and decreased T cell activation and cytotoxicity (Fig. 5P). Importantly, KMT9 ablation combined with CXCR2 inhibition or ARG1 inhibition results in increased inhibition of prostate tumour growth. Taken together, our findings pave the way for the development of novel immunotherapeutic strategies for the treatment of PCa.

## Discussion

Acquired resistance to anti-androgen therapy is a frequently observed severe challenge in PCa patients [1]. Unfortunately, alternative treatment options such as immunotherapy have typically shown low clinical efficacy due to an immunosuppressive TIME [38–41]. Immune checkpoint inhibitors, for example, failed to elicit successful responses in PCa patients [38–40]. The only FDA-approved immunotherapeutic medication for CRPC is Sipuleucel-T [42, 43], however, there have been controversies concerning its benefit [44]. Consequently, in order to develop novel therapeutic options, recent research has aimed to identify strategies to convert the immunosuppressive TME of PCa to an immunoresponsive state.

By using the CXCR2 inhibitor SB255002, Wang et al. showed that CXCL5-CXCR2 signalling pathway is critical for the recruitment of MDSCs and PCa progression [18]. Similarly, a recent study showed that treatment of patients with a combination of the AR inhibitor enzalutamide and the CXCR2 inhibitor AZD5069 resulted in durable clinical benefit in a subset of mCRPC patients [13]. Therapeutic success correlated with a reduction in circulating neutrophils and, in consequence, lower intra-tumour myeloid infiltration [13]. In our study, we uncovered that KMT9 regulates CXCL-CXCR2 signalling by controlling expression of tumour-secreted myeloid chemoattracting CXCR2 ligands such as CXCL2 and CXCL5, and thereby supports the infiltration of PMN-MDSCs in the prostate TIME. PMN-MDSCs distribute around tumour glands to potentially protect prostate tumour cells from immune surveillance by excluding cytotoxic T cells from tumour glands and by restricting their access to tumour cells. Peritumoural distribution of neutrophils has been previously observed in the stroma of solid tumours and linked to poor survival [45]. Our findings indicate that the abundant production of CXCR2 ligands by KMT9α-proficient tumours limits the efficacy of SB225002, consistent with its mechanism of action as a competitive CXCR2 antagonist. In contrast, KMT9α deletion lowers the production of multiple CXCR2 chemokines, thereby reducing the chemokine burden and rendering the residual CXCR2-dependent signalling more susceptible to pharmacological inhibition. Importantly, these in vitro functional data closely recapitulate the in vivo responses to SB225002 and provide a mechanistic explanation for why PMN-MDSC infiltration is not significantly affected in *Pten/Trp53* KO tumours while being substantially more sensitive to CXCR2 blockade in the KMT9α-deficient setting.

Importantly, the immune-excluded phenotype commonly found in solid tumours has been shown to be one of the main reasons for immunotherapy failure [46]. We found that targeting KMT9 not only reduced the infiltration of PMN-MDSCs but also increased the number of cytotoxic T cells in contact with tumour cells, which is critical for the success of T cell-based immunotherapies [5, 7].

We also showed that KMT9 is key to escaping immune surveillance in prostate tumours, since it hampers T cell killing activity. Mechanistically, our data provide evidence that KMT9 controls the expression of ARG1 which is critical to alter cytotoxic T cell killing activity. Arginases, are commonly overexpressed in different types of tumours and have been shown to be upregulated upon *Pten* deletion [47–52]. High ARG1 levels typically correlate with immunosuppression due to impaired T cell proliferation and activity [53, 54]. Accordingly, potent arginase inhibitors such as numidargistat have been developed [55, 56]. However, clinical trials involving arginase inhibitors only showed limited antitumour activity [57], which might highlight the limitation of immunotherapies in contexts where there is poor cytotoxic T cell infiltration within the TME.

Since T cells are excluded from glands of *Pten/Trp53* KO prostate tumours, we hypothesized that ARG1 inhibition would not impact tumour progression in these animals. Indeed, we found that numidargistat treatment of *Pten/Trp53* KO mice increased the activation of T cells but failed to affect tumour growth. These findings might explain why clinical trials using checkpoint inhibitors in PCa with an immune-excluded phenotype have not achieved the expected efficacy [3, 4]. In contrast, numidargistat treatment in *Pten/Trp53/Kmt9α* KO mice with an immune-inflamed phenotype showed a significant impact on tumour regression compared to vehicle. The efficacy of numidargistat after KMT9 deletion suggests a context-dependent vulnerability where tumours become functionally more dependent on remaining ARG1 activity from infiltrating PMN-MDSC ARG1^+^ cells that escapes genetic tumour-cell targeting once other KMT9-dependent immunosuppressive pathways, such as CXCR2 ligand production, PMN-MDSC recruitment and T cell exclusion, have been attenuated.

In summary, our findings identify KMT9 as a critical factor for the establishment of an immune-excluded phenotype in PCa, paving the way for the development of novel immunotherapeutic strategies for the treatment of PCa.

## Methods

### The cancer genomes atlas data analysis

The Cancer Genome Atlas Prostate Adenocarcinoma (TCGA-PRAD, https://portal.gdc.cancer.gov/) was used to assess expression levels of *KMT9α* and *KMT9β* in normal prostate and PCa samples. RNA sequencing data, specifically FPKM (fragments per kilobase million) normalized expression values, were used and downloaded from the TCGAbiolinks R package [58]. Paired Student's t-test was used to compare the expression values of *KMT9α* and *KMT9β* in 52 normal prostate samples and 500 PCa samples. Data of a total of 500 PCa patients with clinical features (age, laterality, topography, lymph node, metastasis) were downloaded from the TCGAbiolinks R package. TCGA survival data were downloaded from TCGA Pan-Cancer Clinical Data Resource (TCGA-CDR) [59]. The Univariate Cox regression analysis was used to calculate the hazard ratios (HR), confidence intervals (CI) of HR, and p values of *KMT9α* (Fig. 1B) and *KMT9β* (Supplemental Fig. 1C). For differential expression analysis (Fig. 1C), PCa samples were divided into high- and low- *Kmt9α* groups according to the median of *Kmt9α* expression values. The analysis was performed on the expression matrix of the samples using the R package edgeR. The screening conditions for differentially expressed genes were: |log_2_Fold Change|> 0.2, p values < 0.05. Heatmaps of differential gene expression (Fig. 1C) were drawn using the R packages ggplot2 and pheatmap. To generate Supplemental Fig. 1D, significant copy number gain or loss in the regions containing indicated genes were assigned by the top and low scores in UCSC Xena platform for PRAD CNV data (cutoff for *PTEN*: ≤ −0.1223 or ≥ 0.2350, *KRAS* ≤ −0.1299 or ≥ 0.1454, *SMAD4* ≤ −0.1227 or ≥ 0.1187, TP53 ≤ −0.4119 or ≥ 0.1187). The remaining data were used as control group.

### Western blot analysis

Experiments were essentially performed as previously described [60]. The Precision Plus Protein™ Kaleidoscope™ Standards (Bio-Rad) was used as size marker. The electrophoresis was performed using a standard Tris–Glycine-SDS running buffer (25 mM Tris, 50 mM Glycine, 0.1% SDS). The specific acrylamide percentage (%) of the SDS-PAGE resolving gel used for each individual western blot experiment is indicated in Supplemental Fig. 6. Prostate tissues were minced and disrupted in RIPA buffer (1 mM EDTA, 50 mM Tris–HCl pH7.5, 0.1% SDS, 150 mM NaCl, 1% NP40, 1% NaDeoxycholate) using the Precellys Lysing Kit (Bertin Technologies) and a Minilys homogenizer (Bertin Technologies). After centrifugation for 10 min at 14,000 rpm, supernatant was collected and protein concentration was determined. The following antibodies were used for Western blot analysis: anti-KMT9α (#228,445, lot: 27,112,018, Schüle Lab, 1/400); anti-GAPDH (Milipore, #MAB574, lot: 3,688,965, 1/500); anti-TRP53 (Linaris, #LIN-P953, lot: 6,065,476, 1/1000); anti-PTEN (Cell Signalling Technology, #9552, lot: 309/2019, 1/1000); anti-beta-Actin (Sigma, #A1978, lot: 0000086304, 1/10000); anti AKT1 (Cell Signalling Technology, #2938, lot: 4, 1/1000); anti-AKT1 S473ph (Cell Signalling Technology, #9018, lot: 5, 1/1000); anti-TUBULIN (Sigma, #T6074, lot: 0000127282, 1/10000); anti-ARG1 (Cell Signalling Technology, #93,668, lot: 4, 1/1000). Chemoluminescent signals were recorded with an Amersham imager 600 (GE Healthcare).

### Mouse chemokine expression profile analysis

The proteome profiler Mouse Chemokine Array Kit (R&D systems) was used to profile chemokine expression in lysates of Ctrl, *Pten/Trp53* KO, *Pten/Trp53/Kmt9α* KO, and *Pten/Trp53/Kmt9α* KO/NLS-KMT9*α* mouse prostates or mouse prostate luminal cells. Experiments were performed following the manufacturer’s instructions. Briefly, the arrays were blocked for one hour in Array Buffer 6 at RT. Then, array membranes were incubated over night at 4 °C with a mix containing 200 µg prostate tissue or cell lysate, 500 µl Array Buffer 4, 1 ml Array Buffer 6, and 15 µl Detection Antibody Cocktail and washed three times in 20 ml Wash Buffer. Membranes were then incubated for 30 min at RT with Streptavidin-HRP diluted 1/2000 in Array Buffer, washed three times 10 min in 20 ml Wash Buffer. Chemoluminescent signals were revealed using the Chemi Reagent Mix and recorded with an Amersham imager 600.

### RNA sequencing (RNA-seq)

RNA from mouse prostate was isolated using the RNeasy Plus Mini kit (Qiagen) essentially as described by the manufacturer. RNA samples were sequenced using the standard Illumina protocol generating raw sequence files (.fastq files) by Novogene UK Ltd. Reads were aligned to the mm10 build of the murine genome using STAR version 2.7.10b. The aligned reads were counted with the Homer 4.11 software with default settings (analyzeRepeats) and differentially expressed genes were identified using EdgeR 2.38.4. Heatmaps were generated using Morpheus (https://software.broadinstitute.org/morpheus/). Data are deposited under GSE253018.

### Isolation and culture of mouse prostate luminal cells

Mouse prostate organoids were obtained essentially as previously described [61]. Briefly, prostates of Ctrl, Pten^fl/fl^/Trp53^fl/fl^, Pten^fl/fl^/Trp53^fl/fl^/Kmt9α^fl/fl^ mice were isolated, and dissociated to obtain a single cell suspension. Then, cells were incubated and labelled with anti-CD45 (eBioscience #11–0451-82; 0.5 µg/100 µl), anti-CD31 (eBioscience #11–0311-82;1 µg/100 µl), anti-Ter-119 (eBioscience #11–592,185; 0.25 µg/100 µl), anti-EpCAM (Biolegend #118,217; 0.25 µg/100 µl), anti-CD49f (Biolegend #313,611; 0.5 µg/100 µl) antibodies, and DAPI (1 µg/ml). Mouse prostate luminal cells (DAPI^−^, Lin^−^(CD45^lo^, CD31^lo^, Ter119^lo^), EpCAM^hi^, CD49^mi^) were then isolated by Fluorescence-activated Cell Sorting (FACS). The single cells obtained were resuspended in a solution containing Matrigel Basement Membran Matrix (Corning #354,234) and Advanced DMEM/F-12 medium (Thermo Fisher Scientific) in a 60:40 ratio. For each dome, approximately 5,000 cells were seeded in a 50 μl drop of Matrigel/Advanced DMEM-F12 in 24-well plates. The Matrigel was allowed to polymerize at 37 °C for 30 min and then covered with 1 ml of Advanced DMEM/F-12 culture medium supplemented with HEPES 10 mM, B-27 1x, GlutaMAX 1x, N-acetyl-L-cysteine 1.25 mM, murine EGF 50 ng/µl, murine Noggin 100 ng/ml, murine R-Spondin-1 500 ng/ml, A83-01 200 nM, DHT 1 nM. Y-37632 10 µM was only added during establishment of culture and passaging of organoids. To generate *Pten/Trp53* KO and *Pten/Trp53/Kmt9α* KO organoids, Pten^fl/fl^/Trp53^fl/fl^ and Pten^fl/fl^/Trp53^fl/fl^/Kmt9α^fl/f^ organoids were dissociated in TrypLE Express Enzym (1x) (Gibco #12,605,036). Then, single cells were infected with Adeno-Cre-GFP-expressing adenovirus (Vector Biolabs) and organoids (10.000 cells/dome) were allowed to grow for three days. Then, upon dissociation, GFP positive cells were FACS-isolated and the obtained single cells were resuspended in a solution containing Matrigel Basement Membran Matrix and Advanced DMEM/F-12 medium in a 60:40 ratio. Domes were covered with Advanced DMEM/F-12 culture medium supplemented with HEPES 10 mM, B-27 1x, GlutaMAX 1x, N-acetyl-L-cysteine 1.25 mM, murine EGF 50 ng/µl, murine Noggin 100 ng/ml, murine R-Spondin-1 500 ng/ml, A83-01 200 nM, DHT 1 nM, Y-37632 10 µM.

To grow Ctrl, *Pten/Trp53* KO and *Pten/Trp53/Kmt9α* KO cells in 2D, organoids were dissociated and single cells were allowed to grow in Advanced DMEM/F-12 medium supplemented with HEPES 10 mM, B-27 1x, GlutaMAX 1x, N-acetyl-L-cysteine 1.25 mM, murine EGF 50 ng/µl, murine Noggin 100 ng/ml, murine R-Spondin-1 500 ng/ml, A83-01 200 nM, DHT 1 nM, Y-37632 10 µM. To generate *Pten/Trp53/Kmt9α* KO/NLS-KMT9α KO cells, *Pten/Trp53/Kmt9α* KO were transduced with pLenti-NLS-KMT9α.

### RNA preparation for quantitative RT-PCR analysis

RNA from mouse prostate was isolated using the RNeasy Plus Mini kit (Qiagen) essentially as described by the manufacturer. Quantitative RT-PCR was performed using the Abgene SYBR Green PCR kit (Invitrogen) according to the supplier’s protocol. *Polr2a* 5’-caccccagcttctcccaaat-3’ and 5’-agtatgtcggggaggttgga-3’ was used for normalization. The following primers were used: *Kmt9*α 5’-cagccgcatgtaccttggaa-3’ and 5’-tccacgacttcctacctcttca-3’; *Krt4* 5’-ctgagggactaccaggctct-3’ and 5’-cagaaccggagcaaaaaccg-3’; *Krt5* 5’-aacgtcaagaagcagtgtgc-3’ and 5’-tccagctctgtcagcttgtt-3’; *Krt6a* 5’-ctgaggaacatgcaggacac-3’ and 5’-ctacatccttcttcagggtcacg-3’; *Krt7* 5’-cgcaacacccggaatgagat-3’ and 5’-tggactctaacttggcacgc-3’; *Krt8* 5’-gtgaaccagagcctgttgag-3’ and 5’-gttgtccatgttgctcctcg-3’; *Krt14* 5’-agcggcaagagtgagatttct-3’ and 5’-cctccaggttattctccaggg-3’; *Krt15* 5’-gctggcttacctgaagaagaacc-3’ and 5’-tcatactgctccctcatctctgc-3’; *Krt16* 5’-tgagctgaccctgtccaga-3’ and 5’-ctcaaggcaagcatctcctc-3’; *Krt19* 5’-ccctcccgagattacaaccac-3’ and 5’-ggcgagcattgtcaatctgt-3’; *Cxcl1* 5’-ccacccgctcgcttctctg-3’ and 5’-tcttgaggtgaatcccagccat-3’; *Cxcl2* 5’-ctgccaagggttgacttcaa-3’ and 5’-ttttgaccgcccttgagagt-3’; *Cxcl3* 5’-tgcctgaacaccctaccaag-3’ and 5’-ggcaaacttcttgaccatcct-3’; *Cxcl5* 5’-ctcagtcatagccgcaacgg-3’ and 5’-ggatccagacagacctccttc-3’; *Arg1* 5’-ctggcctttgttgatgtccc-3’ and 5’-gagatgcttccaactgccag-3’. Custom primers were obtained from Merck.

### Haematoxylin and immunohistochemical staining

Prostate sections were harvested, fixed in 10% formalin for 24 h, washed twice in PBS, and embedded in paraffin. Paraffin blocks were sliced into 5 μm thick sections and slides were stained using a standard procedure for haematoxylin staining. For immunohistochemical staining, slides were deparaffinized with xylene, rehydrated in a descending alcohol series, and finally washed with PBS and H_2_O. Antigen retrieval was performed by pressure cooking in 20 mM citrate buffer (pH 6.0), and the samples were incubated with 3% H_2_O_2_ for 30 min to reduce endogenous peroxidase activity. After blocking for 1 h in PBS containing 3% milk and 0.1% Tween®20, samples were incubated overnight at 4 °C with anti-KMT9α (#28,445, lot 27,112,018, Schüle Lab, 7.5 μg/ml), or IgG control (#Kch-504–250, Diagenode, RRID:AB_2722554, 7.5 μg/ml). For immunohistochemistry, slides were washed and primary antibodies were detected using Vectastain ABC KIT, Rabbit IgG (#PK-4001, Vector Laboratories, RRID:AB_2336810, 7.5 μg/ml) according to the manufacturer’s instructions. Peroxidase substrate (Vector NovaRED, #SK-4800, Vector Laboratories, RRID:AB_2336845) was added, and the slides were washed with H2O, counterstained with haematoxylin and then mounted with Fluoromount-G Mounting Medium (Southern Biotech).

### Mouse studies

PSA-Cre-ER^T2(Tg/0)^/Pten^fl/fl^/Trp53^fl/fl^, PSA-Cre-ER^T2(Tg/0)^/Pten^fl/fl^/Trp53^fl/fl^/Kmt9α^fl/fl^, PSA-Cre-ER^T2(Tg/0)^/Kmt9α^fl/fl^, PSA-Cre-ER^T2(Tg/0)^ mice were used for in vivo experiments. All mice were housed in the pathogen-free barrier facility of the University Medical Center Freiburg in accordance with institutional guidelines and approved by the regional board (Regierungspräsidium Freiburg, 35–9185.81/G-19/26). Mice were maintained under temperature- and humidity-controlled conditions with a 12-h light/dark cycle, free access to water, and a standard (3807, granovit, Kaiseraugust, Switzerland) or 400 mg/kg tamoxifen citrate-containing (A115T70400, ssniff, Soest, Germany) rodent chow. Mice were sacrificed 10 weeks (Fig. 1E) and 8 weeks following tamoxifen-induced gene loss (Fig. 3J and 5K). Animals were killed by cervical dislocation and tissues immediately processed for further analyses.

### Generation of prostate single cell suspension and isolation of CD45 + leukocytes from mouse prostate and blood

Mouse prostates were cut into small pieces and enzymatically digested in MACS C Tubes (Miltenyi Biotec) containing DMEM (Gibco), Enzyme D, R, A (Tumor Dissociation Kit mouse; Miltenyi Biotec) and DNase I (Roche) using the MACS Octo Dissociator (Miltenyi Biotec) with heaters. To further disaggregate the tissue, samples were passed through a 20G needle. Cell suspensions were then filtered through a 70 μm strainer (Corning) and subsequently through a 30 μm strainer placed on a Falcon tube. Samples were centrifuged at 300 × g for 7 min, the supernatant was removed, and cells were resuspended in DMEM and counted after staining with Trypan blue (Sigma). For the isolation of prostate CD45^+^ infiltrating leukocytes, cells were magnetically labelled with CD45 MicroBeads (Miltenyi Biotec) and separated using MACS columns and a MACS separator (Miltenyi Biotec). Leukocytes from mouse peripheral blood were obtained by cardiac puncture. Erythrocytes were removed by incubating blood with ammonium chloride lysis buffer (ACK Lysing buffer, ThermoFisher) for 5 min at RT. Cells were washed with cold PBS and anti-CD45 antibody was included in subsequent flow cytometry analysis to discard any residual non-hematopoietic cell.

### Single-cell spatial phenotyping

Multiplex staining was performed using the PhenoCycler®-Fusion system essentially as described in the Phenocycler-Fusion User Guide from Akoya. 5 µm thick FFPE tissue sections were fixed on poly-L-lysine-coated slides (Leica Apex Superior Adhesive Slides, #350,080), dried over night at room temperature, and incubated on a heating plate at 65 °C to allow paraffin melting. Deparaffinization and hydration of samples was performed by successive 5 min incubations in HistoChoiceClearing Agent (VWR #H103), twice in 100%, 90%, 70%, 50%, and 30% ethanol, and final washing with ddH_2_O. Antigen retrieval was performed by cooking samples for 20 min in a pressure cooker in 1 × AR solution (Akoya) followed by cooling down to room temperature for 30 min, and two washing steps in ddH_2_O. Then, samples were incubated twice in Hydration Buffer (Akoya) for 2 min and incubated in Staining Buffer (Akoya) for 30 min. Antibody Cocktail Solution containing N, G, J, and S Blocker (Akoya) and primary antibodies (Supplemental Table 1) were prepared and applied to the sample slide. HybriSlides (Grace Biolabs #714,022) were gently placed on top and incubated over night at 4 °C. The next day, slides were washed twice for 2 min in Staining Buffer. The sample were fixed in Post Staining Fixing Solution (Akoya; PFA (16%)) in Storage Buffer (1:9 v/v) for 10 min at room temperature and washed three times in PBS. For the second fixation step, slides were incubated in ice-cold methanol for 5 min and washed three times in PBS. For the last fixation step, 200 µl per slide of Final Fixative Solution (Akoya) were added, incubated for 20 min at room temperature, washed three times in PBS, and stored in Storage Buffer (Akoya). Phenocycler Reporter Plate was prepared based on the total number of cycles (including two blank cycles). 245 µl per well of Reporter Stock Solution (Akoya, 1 × Buffer for PhenoCycler with Buffer Additive, Assay Reagent, Nuclear stain) were pipetted into the blank wells. Reporter Master Mix was prepared with 5 µl of each Reporter (Supplemental Table 1) and 250 µl Reporter Stock Solution, and added into a 96 well plate. The plate was sealed and a fourteen cycle Phenocycler program was performed essentially as described by the manufacturer. Briefly, the workflow consisted of iterative cycles of labelling, imaging, and removal of fluorescent reporters. During each imaging cycle, three fluorescent reporters were assayed according to their corresponding barcode-conjugated antibodies and imaged via epifluorescence optics. Thereafter, the three reporters were removed, and a new cycle started during which additional reporters were imaged.

The computational analysis workflow for phenotyping included a quality control assessment of each marker, followed by cell segmentation based on nuclear DAPI staining using the QuPath software (v0.4.3) [62]. The average marker intensities were calculated for each cell from the nuclear expression compartment. The resulting expression table was exported to R/Seurat-package. Clustering for phenotyping was performed according to the Seurat standard protocol. Briefly, the imported data were clustered (FindClusters, FindAllMarkers) to find differentially expressed markers (min.pct = 0.25, logfc.threshold = 0.25) in each cluster. To determine accurate cell type annotations based on staining profiles, clusters were classified and merged resulting in the identification of the indicated cell types. Resulting annotations were reimported to QuPath for quantification and visualization.

### Flow cytometry

Isolated CD45^+^ infiltrating leukocytes were incubated with TruStain FcX (anti-mouse CD16/32 antibody, Biolegend) and Zombie NIR (Biolegend) for 20 min at room temperature in the dark to block Fc receptors and to exclude dead cells, respectively. Cells were washed with 1 × PBS, 0.5% BSA (Sigma-Aldrich) (cytometry staining buffer, CSB). Cells were then fixed with eBioscience Foxp3/Transcription Factor Staining Buffer Set (Invitrogen) for 1 h and washed twice with CSB. All samples were incubated for 20 min at 4 °C in the dark with antibody master mix, washed with CSB and centrifuged at 300 × g for 5 min at 4 °C. For the staining of intracellular epitopes, cells were permeabilized with eBioscience Foxp3/Transcription Factor Staining Buffer Set (Invitrogen) for 30 min at RT in the dark. Then, cells were washed with permeabilization buffer (Invitrogen) and centrifuged at 300 × g for 5 min at 4 °C. The supernatant was discarded and cells were incubated for 30 min at RT in the dark with antibody master mix. After washing cells with CSB, cells were acquired with a BD LSRFortessa cytometer (BD Bioscience) and analysed by FloJo software (BD Bioscience). The following antibodies obtained from Biolegend were used for staining: anti-CD45-AF700 (#103,128, lot: B348053, 1/200); anti-CD11b-APC (#101,211, lot: B374880, 1/80); anti-Ly6G-Pacific blue (#127,612, lot: B380041, 1/50); anti-Ly6C-BV785 (#127,612, lot: B347927, 1/160); anti-CD3-BV785 (#127,612, lot: B365876, 1/40); anti-CD4-PerCP Cy5.5 (#100,434, lot: B314210, 1/80); anti-CD25-BV711 (#1,102,049, lot: B359380, 1/160); anti-CD8-PE/Dazzle 594 (#100,762, lot: B317390, 1/160); anti-EpCAM-APC (#118,214, lot: B280290, 1/80); anti-EpCAM-FITC (#118,208, lot: B288878, 1/200); anti-Granzyme B- PE/Cy7 ((#396,410, lot: B352429, 1/20); Zombie NIR-APC Cy7 (#423,106, lot: B354830, 1/500).

### Single-cell mRNA sequencing (scRNA-seq)

Murine prostate tumour samples were micro dissected and isolated from surrounding tissue, flushed in cold PBS and kept on ice in DMEM/F-12 media. Subsequently, respective tissues were transferred into pre-cooled tissue preservation solution (Singleron Biotechnologies GmbH, Germany) after a second round of flushing in ice-cold PBS. Five tumour samples were pooled per group. Prostate single cell suspensions were counted using a LUNA automated cell counter (Logos Biosystems). Single cell capture, reverse transcription, and library preparation were carried out on the Chromium platform (10 × Genomics) with the singlecell 3′ reagent v2 protocol according to the manufacturer's recommendations using 1 × 10^4^ cells as input per reaction well. The two final libraries *Pten/Trp53* KO and *Pten/Trp53/Kmt9α* KO were pooled and sequenced on two Illumina NovaSeq SP lanes (paired-end 26 bp + 96 bp). Raw sequencing data were processed and aligned to the mouse genome (mm10) using the CellRanger pipeline (10 × Genomics version 3.1, SCR_017344). Filtered raw-count matrices were further analysed using the Seurat (v3) R package for data integration and unbiased clustering of single cell transcriptomes. Previously published scRNA-seq data from mouse prostate (GSE146811) and sequenced mouse prostate from *Pten/Trp53* KO and *Pten/Trp53/Kmt9α* KO mouse strains were analysed together. Low-quality clusters with low numbers of detected genes and high percentages of mitochondrial genes that lacked expression of biologically relevant cell type markers were removed after initial clustering (300 < nFeature_RNA < 5000, percent.mt < 30). Datasets were normalized independently using the SCTransform method. Technical (percent.mt, nFeature_RNA) were regressed out using the var.to.regress argument in the SCTransform function. Both datasets were harmonized using canonical correlation analysis (SCT) with default parameters. Principal component analysis (PCA) was used for dimensionality reduction and the K-nearest neighbour (KNN) graph was constructed using the first 30 principal components (PCs). Cells were clustered using the Louvain algorithm and embedded in two-dimensional space (30 PCs) using Uniform Manifold Approximation and Projection (UMAP). Differentially expressed genes between clusters were identified using Wilcoxon Rank Sum test and clusters were assigned to four major cell types according to expressed marker genes and their similarity to prostate cell types. We merged clusters if their average gene expression profiles were highly correlated and if they were characterized by similar cell type-specific marker genes. Plots were generated with Seurat, genesorteR or custom R script using ggplot2. Data are deposited under GSE253018.

### Chromatin immunoprecipitation and sequencing (ChIP-seq)

ChIP experiments were performed essentially as previously described [56]. *Pten/Trp53* KO mouse prostate luminal cells were cultured as described above. Immunoprecipitation was performed with specific antibodies (anti-KMT9α (#2G7, lot 21,940–052824-F01, Schüle Lab) on GammaBind^TM^G-Sepharose™ (GE-Healthcare). Libraries were prepared from immunoprecipitated DNA according to standard methods. ChIP-seq libraries were sequenced using a NovaSeq 2000 (Illumina) at Novogene. Reads were aligned to the mm10 build of the mouse genome using Bowtie2 [63]. Data were further analysed using the peak finding algorithm MACS 1.42 [60] using input as control. All peaks with FDR greater than 5% were excluded from further analysis. The reads were used to generate the genome-wide intensity profiles, which were visualized using the IGV genome browser [61]. HOMER [62] was used to annotate peaks (annotatePeaks.pl). The genomic features (promoter, exon, intron, 3’UTR, and intergenic regions) were defined using Refseq. Data are deposited under GSE253018.

### Multiplexed immunofluorescence analysis

Prostate tissues from Ctrl, *Pten/Trp53* KO, and *Pten/Trp53/Kmt9α* KO mouse models were collected and fixed in 4% paraformaldehyde. Sections of 5 μm thickness were obtained using a microtome and mounted onto glass slides. Deparaffinization was achieved by heating slides on a heating plate at 55–60 °C until paraffin melted, followed by sequential incubation in xylene and ethanol gradient. Multiplex immunofluorescence staining was performed using the Opal 7-colour kit (Akoya Biosciences) according to the manufacturer's instructions with slight modifications. Briefly, antigen retrieval was achieved by heating sections in AR6 Buffer in a pressure cooker for 20 min. Next, slides were blocked with Antibody Diluent, then incubated with primary antibodies against the first marker *Kmt9α* (28,445, lot 27,112,018, Schüle Lab; 1/100) overnight at 4 °C, followed by secondary antibody incubation, Opal fluorophore incubation, and antibody removal. Subsequent antibodies against PTEN (9552S, CST, lot 3 08/2019, 1/50), and TP53 (LIN-P956, Linaris, lot 6,065,476, 2.5/1000) were stained using the same protocol. Additional sequential stainings were performed in AR9 Buffer with antibodies against: Ly6G (87048 T, CST, lot 1A8, 6.5/1000), ARG1 (93,668, CST, lot 4, 1/100 in AR6 buffer), CD8a (98941 T, CST, lot 6, 2.5/1000), PD-1 (84651S, CST, lot 7, 1/100), CD69 (10,803–1-AP, Proteintech, lot 00090170, 1/1000), CD8a (98941 T, CST, lot 6, 2.5/1000 in AR6 buffer), PanCK (MSM2-371, NeoBiotech, lot MSM2-371P250220, 35/1000) or arginine (ABIN7439879, lot A20250228114, 1/100, antibodies-online.com). Spectral DAPI staining was conducted after completing all marker stainings. Slides were mounted in Fluoromount and imaged using a PhenoImager™ Fusion (Akoya Biosciences) for image analysis of protein expression and distribution in prostate tissue samples.

### T cell suppression and cytotoxicity assays

T cells were isolated from mice spleens as previously reported [64]. Briefly, spleens were cut into small pieces, smashed against a 70 µm cell strainer using a scrapper, and washed several times with splenocyte isolation buffer (2% FCS, 2 mM EDTA PBS). Then, cells were pelleted and resuspended in ammonium chloride lysis buffer (ThermoFisher) for 5 min at room temperature to lyse red blood cells. Cells were washed with splenocyte isolation buffer, pelleted again, and number of live cells was counted using trypan blue. Isolation of T cells from the cell suspension was performed by using the Pan T cell Isolation kit from Miltenyi according to manufacturer´s protocol and viable isolated T cells were counted using trypan blue.

For T cell suppression assays, T cells were stained with 1 µM CellTrace Violet Cell Proliferation kit (ThermoFisher) for 20 min at 37 °C protected from light. T cells were washed with T cell basal medium (10% FCS, 1% P/S RPMI1640 medium (ThermoFisher), pelleted and incubated for 10 min with T cell basal medium to allow acetate hydrolysis of the dye. Then, T cells were activated for 1 h at 37 °C with Dynabeads (Invitrogen) in a bead-to-cell ratio 1:1. PMN-MDSCs were isolated from mouse prostates using the MACS Tumour Dissociation kit from Miltenyi following manufacturer´s instructions. MDSC Isolation Kit from Miltenyi was used to isolate PMN-MDSCs from prostate cell suspensions following the manufacturer´s instructions. Viable isolated PMN-MDSCs were counted using trypan blue and co-cultured for 3 days with activated T cells in a T cell-PMN-MDSC ratio 1:2. Pictures were taken using the EVOS FLc imaging system (Invitrogen). Beads were removed before staining and the same procedure as detailed above was followed for flow cytometry analysis using the same antibodies with the inclusion of anti-CD69-BV510 (#104,532, Biolegend, lot: B453083, 1/20). Proliferation is represented as mean fluorescence intensity (MFI).

For T cell cytotoxicity assays, a serial two-fold dilution of tumour cell to T cell ratios (1:50, 1:25, 1:12.5, and 1:6.25) was evaluated to identify conditions that provided the best dynamic range for detecting differences in T-cell-mediated cytotoxicity. Thus, T cells were activated for 24 h and then co-cultured with prostate tumour cells in a tumour cell to T cell ratio of 1:6.25 in presence of 10 nM SYTOX Green Nucleic Acid Stain (Invitrogen) to detect tumour cell death in Incucyte S3 Live-Cell Analysis System (Sartorius) for over 4 days. Images were acquired every 6 h and proliferation and cytotoxicity was quantified using Incuyte S3 Software. For ARG1 inhibition experiments, tumour cells were pre-treated with 10 µM numidargistat for 24 h before adding activated T cells and ARG1 inhibition was maintained throughout the entire experiment.

### Mouse neutrophil isolation

Mouse neutrophils were isolated as previously reported [64]. Briefly, mouse tibias and femurs were cut at both ends and bone marrow cells were flushed out with a cold cell suspension buffer (CSB: 1xPBS, 1% FBS, 2 mM EDTA) using a 25-gauge needle and a 5 ml syringe. Cell suspension was then filtered through a 70 µm strainer, washed with CSB and centrifuged at 1.500 rpm at 4 °C for 5 min. Red blood cells were lysed by incubation with ACK lysis buffer (ThermoFisher) for 5 min at RT. Bone marrow cell suspension was then washed with cold CBS buffer and counted using Trypan blue to determine cell viability. Mouse neutrophils granulocytes were isolated by depletion of non-target cells using the neutrophil isolation kit from Miltenyi (Miltenyi Biotec) according to manufacturer´s indications. Viable bone marrow cells were then labelled with a neutrophil Biotin-Antibody Cocktail for 10 min at 4 °C, followed by incubation with anti-Biotin MicroBeads for 15 min and depletion of labelled non-target cells on MACS columns.

### Transwell chemotaxis assays

Migration of bone marrow neutrophils in response to CXCR2 inhibition was assessed by transwell chemotaxis assays similarly as described previously [64, 65]. *Pten/Trp53* KO and *Pten/Trp53/Kmt9α*  KO cells were seeded in full medium and cells were allowed to attach to the plate for 4 h. Then, full medium was replaced for Advanced DMEM/F12 medium to generate *Pten/Trp53* KO and/or *Pten/Trp53/Kmt9α*  KO conditioned mediums (CM) over night. Bottom receiving wells of a 96-transwell plate with 3 µm pores (Corning) were filled with 200 µl of *Pten/Trp53* KO and/or *Pten/Trp53/Kmt9α*  KO CM (1:10 in Advanced DMEM/12). Advanced DMEM/12 and Advanced DMEM/F12 with 100 ng/ml mouse CXCL5 protein (Novus Biologicals) were used as negative and positive controls of migration, respectively. Neutrophils were incubated in Advanced DMEM/F12 with SB225002 (500 nM) for 30 min before being plated in the upper chamber of the transwell plate (500.000 cells/75 ul Advance DMEM/F12 per well). The plate was incubated at 37 °C with 5% CO_2_ for 1 h and migrated neutrophils were counted by taking 10 µl medium from the bottom receiving wells and using an hemocytometer. Total migrated neutrophils were quantified using the formula N = n × 10^4^ × 0.2.

### Tissue microarrays analysis

Human prostate tissue microarrays containing normal prostate, normal adjacent, and PCa tissue were purchased from TissueArray.Com LLC (cat. No.: PR1921c) and utilized for multiplex immunofluorescence staining to assess the expression levels and distribution patterns of KMT9α and ARG1. Staining was performed using the Opal 7-color kit (Akoya Biosciences) following the same protocol as described in the “ Multiplexed immunofluorescence analysis” section. Image analysis was performed using QuPath software. The H-score [66], was utilized to evaluate staining intensity of each marker. For H-score calculations, staining intensities were assigned to four levels: negative, weak, moderate, and strong corresponding to scores of 0, 1, 2, and 3, respectively. Then the percentage of cells at each staining intensity level relative to the total number of cells was determined. The H-score was calculated by multiplying the percentage of cells at each staining intensity level with the corresponding score. The sum these values is represented in a comprehensive assessment of protein expression levels on a scale of 0 to 300.

### Statistics and reproducibility

Statistical analyses were performed using GraphPad Prism. Data are represented as mean + or ± standard deviation (s.d.) as indicated in the figure legends. Differences in sample numbers between experimental groups (Fig. 1E, 1 F, 2A, 2 N, 3L, 5M) or among individual immune cell analyses (Fig. 2B, 3D, 3E, 3G and Supplemental Fig. 2A, B) reflect the availability of independent mouse cohorts used for each experiment and the fact that not all analyses were performed on every cohort. Sample sizes (n) representing individual biological replicates or independent samples are indicated in the corresponding figure legends. Significance was calculated by unpaired Student’s t-test, one-way ANOVA test, two-way ANOVA test, one-sided hypergeometric test, Mantel–Cox survival test, or the Benjamini–Hochberg procedure as indicated. No statistical method was used to predetermine sample size. No data were excluded from the analyses. The experiments were not randomized, and the investigators were not blinded to allocation during experiments and outcome assessment. Sample sizes and p-values are indicated where appropriate.

## Supplementary Information

Supplementary Material 1: Supplemental Fig. 1: KMT9 controls expression of PMN-MDSC-chemoattractants in PCa. A, Table depicting numbers and clinical features of human normal prostate and prostate tumour samples in the TCGA-PRAD cohort. B, Box-Whisker plots with min to max values* showing KMT9*β mRNA expression in healthy human prostate (blue, *n*=52) and prostate tumour samples (red, *n*=500). P value obtained by unpaired Student’s test. C, Kaplan-Meier progression-free survival analysis for patients with prostate tumours expressing high (red, n=240) or low (blue, n=241) levels of *KMT9*β* mRNA*. HR = hazard ratio. A-C, Data were extracted from TCGA-PRAD. D, Box-Whisker plots with min to max values displaying Kmt9a expression in human prostate tumours with different genetic aberrations. The number of tumour samples (n) is indicated. E, Representative view of Ctrl and Kmt9a KO mice prostates. F-H Ctrl and Kmt9a KO mice prostates were haematoxylin stained (f) or analysed by immunohistochemistry (G) and Western blot (H) using anti-KMT9a antibody. Rabbit (r) IgG (G) and GAPDH (h) served as controls. I, J, Western blot analysis of Pten/Trp53 KO prostate using the indicated antibodies. GAPDH served as control. K, L, Four colour multiplex immunofluorescence staining (K) and box-Whisker plots with min and max values representing signal intensity quantification for the indicated proteins (L) in epithelial cells in Ctrl prostates and tumours cells in Pten/Trp53 KO, and Pten/Trp53/Kmt9a KO mice prostates. Monolayers of epithelial cells in normal prostates (Ctrl) are indicated by arrows. Areas of tumour cells in Pten/Trp53 KO and Pten/Trp53/Kmt9a KO are delimited by dashed line. Scale bars represent 40 μM. P values obtained by one-way ANOVA test are indicated. M-O, Analysis of protein expression of the indicated chemokines (M) by Western blot (N) of Ctrl, Pten/Trp53 KO, and Pten/Trp53/Kmt9a KO mice prostates using a proteome chemokine array. Western blot signal intensities were quantified (O). p, UMAP plot showing the different cell populations identified by scRNA-seq in Ctrl, Pten/Trp53 KO, and Pten/Trp53/Kmt9a KO mice prostates. Q, R, Dot blots showing expression levels of markers used to identify the different cells population in Ctrl, Pten/Trp53 KO, and Pten/Trp53/Kmt9a KO prostates by scRNA-seq. S, Dot blot showing expression levels of Kmt9a in the different cell populations identified by scRNA-seq in Ctrl, Pten/Trp53 KO, and Pten/Trp53/Kmt9a KO prostates. T, Dot plot showing expression levels of Kmt9a in luminal cells of Ctrl, Pten/Trp53 KO, and Pten/Trp53/Kmt9a KO prostates identified by scRNA-seq. U, V, Violin plots showing expression levels of Pten (U) and Trp53 (V) in luminal cells of Ctrl, Pten/Trp53 KO, and Pten/Trp53/Kmt9a KO prostates. W, Loss of genes in Pten/Trp53 KO and Pten/Trp53/Kmt9a KO prostate luminal cells was validated by Western blot using the indicated antibodies. GAPDH and extracts of normal prostate luminal cells (Ctrl) were used as controls. X, diagram showing the number of genes identified by ChIP-seq in Pten/Trp53 KO cells displaying KMT9a localization at gene promoters. Y, Expression of NLS-KMT9a in Pten/Trp53/Kmt9a KO prostate luminal cells transduced with pLenti-NLS-KMT9a was verified by Western blot analysis using the indicated antibodies. a-Tubulin served as control. Z, AA, Analysis of protein expression of the indicated chemokines by Western blot (Z) of Ctrl, Pten/Trp53 KO, Pten/Trp53/Kmt9a KO, and Pten/Trp53/Kmt9a KO/NLS-KMT9a mouse prostate luminal cells using a proteome chemokine array. Western blot signal intensities were quantified (AA). Data represent means +/- s.d. (O, AA). P values obtained by unpaired Student T test (B, D) and by two-way ANOVA test (L) are indicated. Supplemental Fig. 2: KMT9 regulates composition and spatial distribution of immune cell populations within the TIME. A, B, Multiparametric flow cytometry analysis of blood leukocytes of Ctrl, Pten/Trp53 KO, and Pten/Trp53/Kmt9a KO mice. Percentages of blood myeloid cells (CD45^+^ CD11b^+^) (A) and neutrophils (CD45^+^ CD11b^+^ Ly6G^+^) (B) are shown. Data represent means +s.d. n, number of mice analysed. C, D, Representative flow cytometry plots of peripheral blood myeloid cells (C) and neutrophils (D) in Ctrl, Pten/Trp53 KO, and Pten/Trp53/Kmt9a KO mice. E-I, Representative flow cytometry plots of prostate tumour infiltrating myeloid cells (CD45^+^ CD11b^+^) (E), M-MDSCs (CD45^+^ CD11b^+^ Ly6G^-^ Ly6C^hi^) and PMN-MDSCs (CD45^+^ CD11b^+^ Ly6G^+^ Ly6C^lo^) (F), CD45^+^ CD3^+^ CD4^+^ CD25^+^ cells (G), cytotoxic T cells (CD45^+^ CD3^+^ CD8a^+^) (H), activated cytotoxic T cells (CD45^+^ CD3^+^ CD8a^+^ GZMB^+^) (I). J, QRT-PCR analysis showing relative mRNA levels of the indicated genes in Ctrl, Pten/Trp53 KO, and Pten/Trp53/Kmt9a KO mice prostates. Data represent means +s.d. n=3 represents the number of biologically independent samples. K, Cross-correlation matrix for markers and corresponding cell types identified by single-cell phenotyping in Ctrl, Pten/Trp53 KO, and Pten/Trp53/Kmt9a KO prostates. L, UMAP plot showing the cell populations identified by single -cell phenotyping in Ctrl, Pten/Trp53 KO, and Pten/Trp53/Kmt9a KO prostates. M, Dendrogram and unsupervised hierarchical clustering heatmap of cell types identified by single-cell spatial phenotyping in Ctrl, Pten/Trp53 KO, and Pten/Trp53/Kmt9a KO mice prostates. N, Distribution of normal epithelial, tumour epithelial, and proliferating cells in Ctrl, Pten/Trp53 KO, and Pten/Trp53/Kmt9a KO prostates unravelled by single-cell phenotyping. Cell types and antibodies used for staining are indicated. Scale bars represent 100 µm. O, Bar graphs representing distribution and percentages of normal and tumour epithelial cell populations, proliferating cells, and fibroblast in Ctrl (n=5), Pten/Trp53 KO (n=5), and Pten/Trp53/Kmt9a KO (n=3) prostates identified by single-cell spatial phenotyping. n, represents the number of prostate samples analysed. Data represent means +s.d. P values were obtained by one-way ANOVA test (A, B, J) and by two-way ANOVA test (O). Supplemental Fig. 3: Kmt9a loss combined with CXCR2 inhibition increases inhibition of prostate tumour growth. A, Dot blot showing expression levels of Cxcr2 in different cell populations identified in Ctrl, Pten/Trp53 KO, and Pten/Trp53/Kmt9aKO prostates. B, Bar graphs representing distribution and percentages of PMN-MDSCs and CXCR2^+^ PMN-MDSCs in Ctrl, Pten/Trp53 KO, and Pten/Trp53/Kmt9a KO prostates identified by flow cytometry (n=4). n, represent the number of prostates analysed. C, Schematic protocol for treatment of mice with tamoxifen (Tam) and the CXCR2 inhibitor SB225002. D, Overall weight of Ctrl, Pten/Trp53 KO, and Pten/Trp53/Kmt9a KO mice after treatment with SB225002 or vehicle. n=4 mice per condition. Data represent means +/- s.d. E, Representative images of prostates of Ctrl, Pten/Trp53 KO, and Pten/Trp53/Kmt9a KO mice treated with vehicle or SB225002. Prostate structures of Ctrl, Pten/Trp53 KO, and Pten/Trp53/Kmt9a KO mice treated with vehicle or SB225002 revealed by staining with anti-SMA and anti-Pan-KRT antibodies. Scale bars represent 1000 µm. F, Bar graphs representing the percentages of CD8a^+^ T cells in Pten/Trp53 KO and Pten/Trp53/Kmt9a KO prostates of mice treated with vehicle or SB225002 identified by single-cell spatial phenotyping. n, represents the number of prostate samples analysed. Supplemental Fig. 4: KMT9-regulated expression of ARG1 impairs T cell cytotoxicity in PCa. A-F, Growth of Pten/Trp53 KO (A,D), Pten/Trp53/Kmt9a KO (B, E), and Pten/Trp53/Kmt9a KO/NLS-KMT9a (C, F) prostate tumour cells alone or cocultured in presence of autologous (A-C) or syngeneic (D-F) T cells activated with anti-CD3/CD28 beads. n=3 represents the number of biologically independent samples. Data represent means +/- s.d. P values were obtained by two-way ANOVA test. G, ChIP-seq tracks showing KMT9a at representative genes in Pten/Trp53 KO mouse prostate tumour cells. Supplemental Fig. 5: Kmt9a loss combined with ARG1 inhibition induces increased inhibition of prostate tumour growth. A, Immunofluorescence analysis of ARG1 expression using the indicated antibodies. Expression was evaluated in prostate epithelial cells from Ctrl, Pten/Trp53 KO, and Pten/Trp53/Kmt9α KO prostates, as well as in PMN-MDSCs and CD8a+ T cells. B, Schematic protocol for treatment of mice with tamoxifen (Tam)-containing diet and the ARG1 inhibitor numidargistat. C, Overall weight of Pten/Trp53 KO and Pten/Trp53/Kmt9a KO mice after treatment with numidargistat. n=3 mice per condition. Data represent means +/- s.d.

Supplementary Material 2: Supplemental Fig. 6: Source data for western blots shown in Fig. 5F, Supplemental Fig. 1H, 1L, 1J, 1W, 1Y.

Supplementary Material 3.

## Data Availability

The authors declare that the data supporting the findings of this study are available within the article and its Supplemental Data Supplemental Information files. RNA-seq, scRNA-seq and ChIP-seq data have been deposited at GEO under GSE253018.
