# Supplementary material for: KMT9 drives T cell exclusion and dysfunction by promoting PMN-MDSCs infiltration and ARG1 expression in prostate cancer
Source: Mol Cancer. 2026 Sep 17;25:223. doi: 10.1186/s12943-026-02801-8 (PMC13587405; doi:10.1186/s12943-026-02801-8)

Supplemental Fig. 1: KMT9 controls expression of PMN-MDSC-chemoattractants in prostate cancer

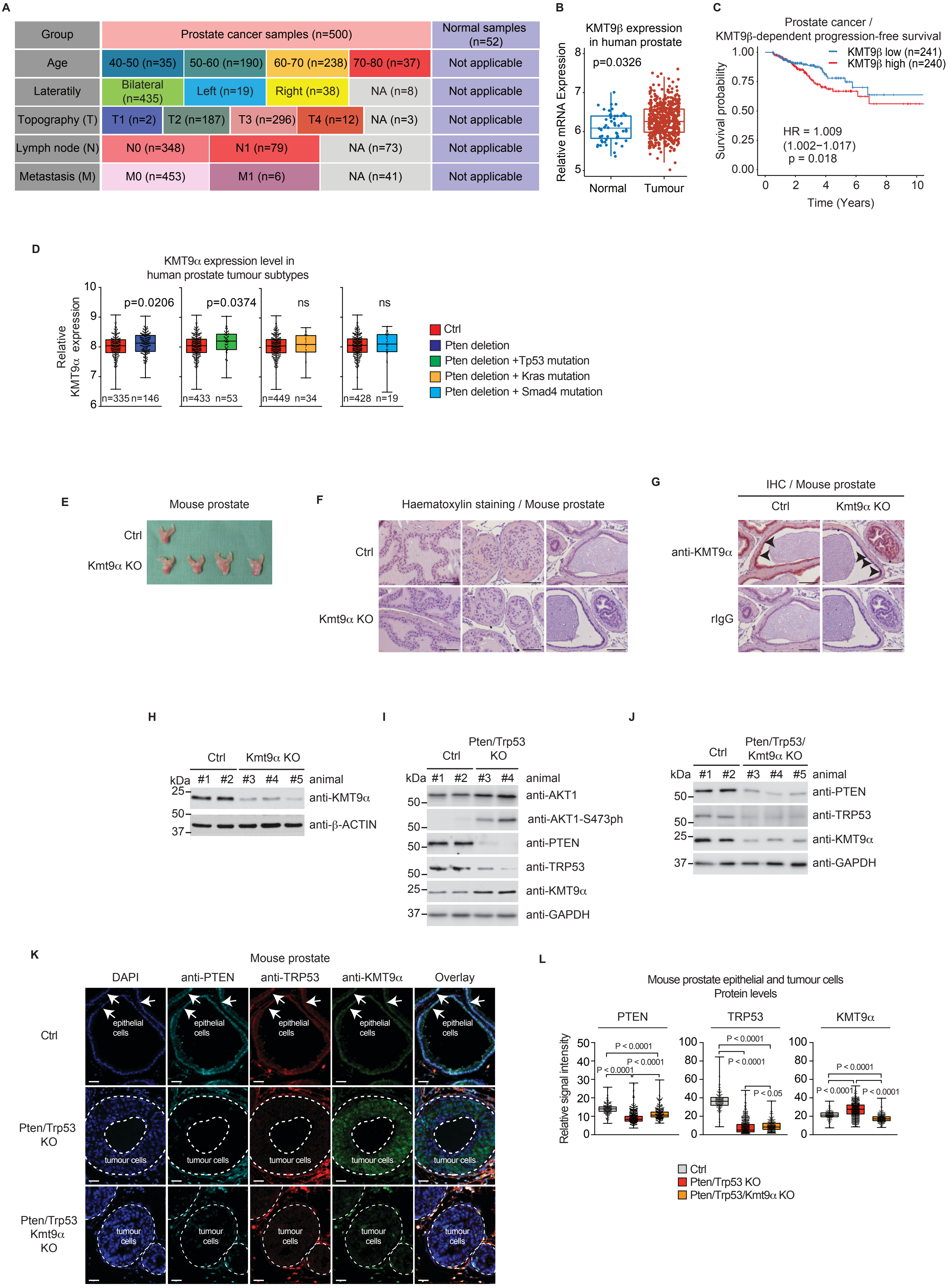

Group	Prostate cancer samples (n=500)					Normal samples (n=52)
Age	40-50 (n=35)	50-60 (n=190)	60-70 (n=238)	70-80 (n=37)		Not applicable
Lateratily	Bilateral (n=435)	Left (n=19)	Right (n=38)	NA (n=8)		Not applicable
Topography (T)	T1 (n=2)	T2 (n=187)	T3 (n=296)	T4 (n=12)	NA (n=3)	Not applicable
Lymph node (N)	N0 (n=348)	N1 (n=79)	NA (n=73)			Not applicable
Metastasis (M)	M0 (n=453)	M1 (n=6)	NA (n=41)			Not applicable

Supplemental Fig. 1: KMT9 controls expression of PMN-MDSC-chemoattractants in prostate cancer

M

Mouse chemokine array coordinates

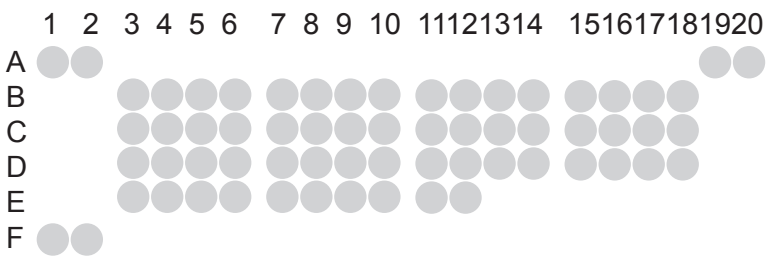

B	3	4	CCL21
B	5	6	CXCL13
B	7	8	CCL6
B	9	10	C5/C5a
B	11	12	CCL28
B	13	14	RARRES2
B	15	16	CCL27
B	17	18	CXCL16
C	3	4	CCL11
C	5	6	CXC3CL1
C	7	8	IL16
C	9	10	CXCL10
C	11	12	CXCL11
C	13	14	CCL2
C	15	16	CXCL1
C	17	18	CXCL5
D	3	4	CCL8
D	5	6	CCL12
D	7	8	CCL22
D	9	10	CXCL9
D	11	12	CCL3/4
D	13	14	CCL9/10
D	15	16	CXCL2
D	17	18	CCL5
E	3	4	CXCL12
E	5	6	Complement Factor D (Ctrl)
E	7	8	gp130 (Ctrl)
E	9	10	HSP60 (Ctrl)
E	11	12	Negative Ctrl

N

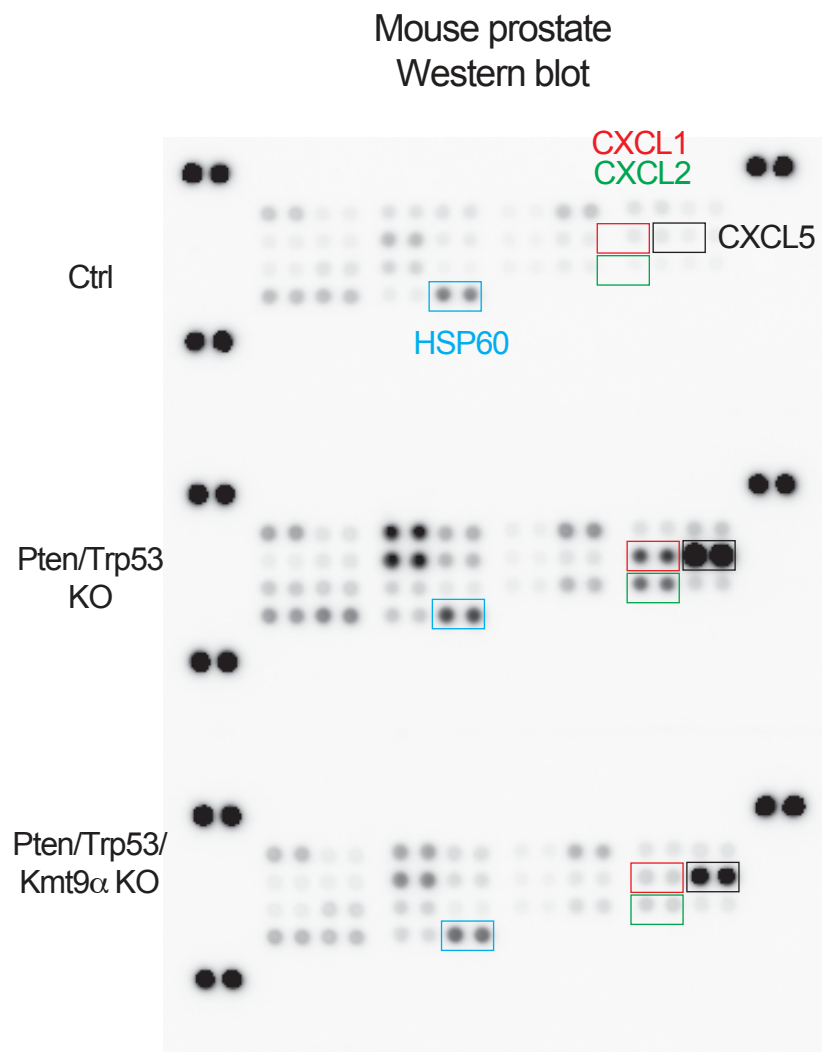

O

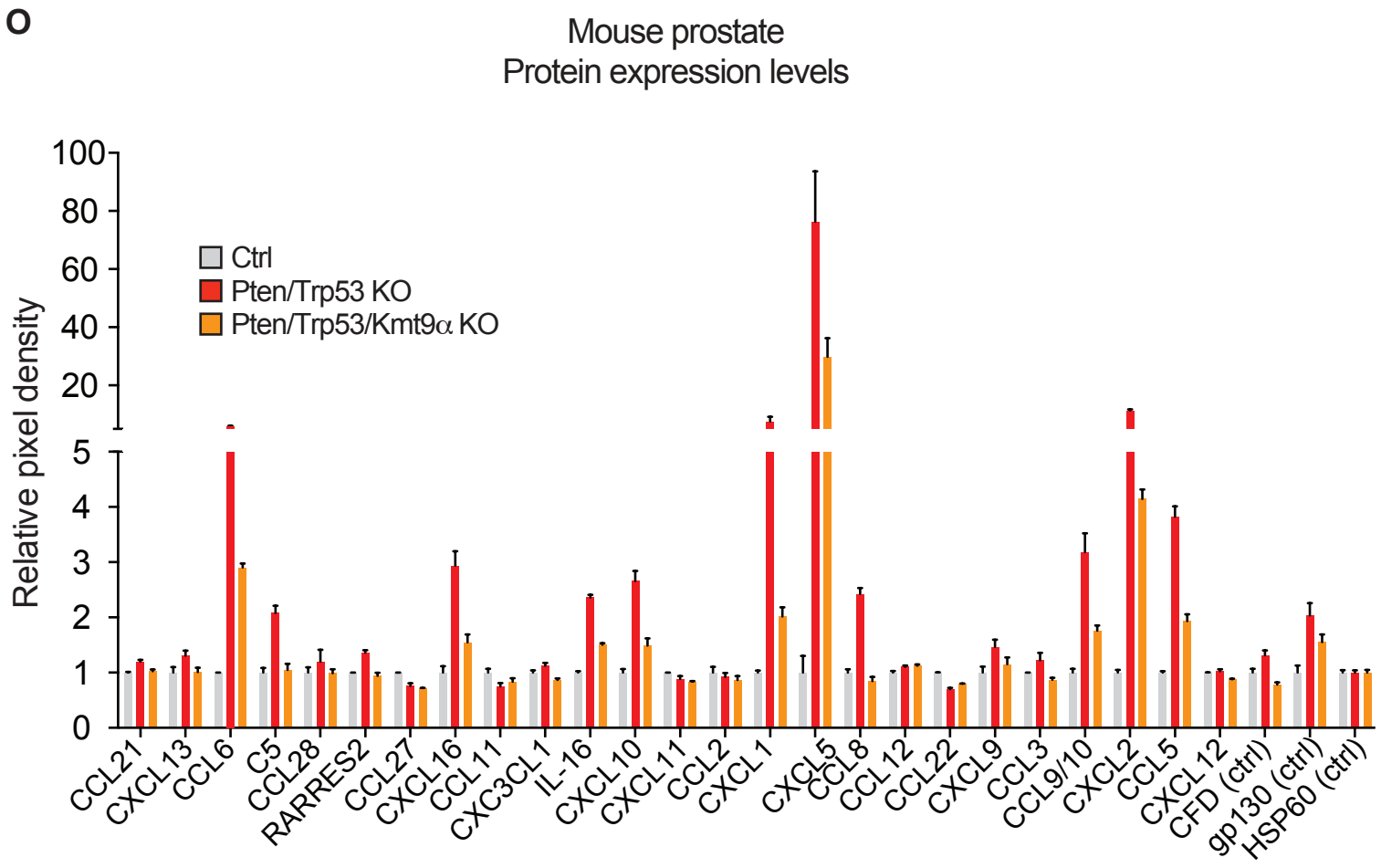

P

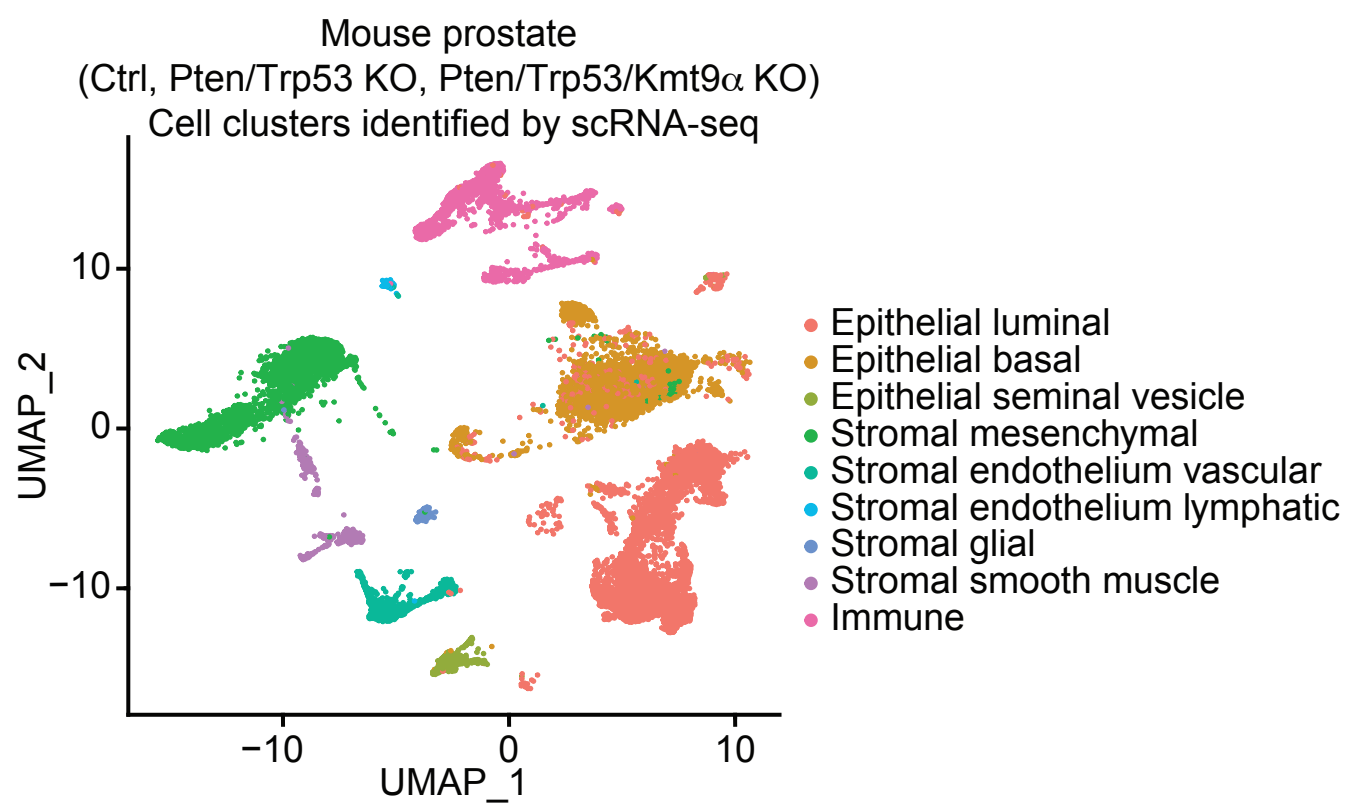

Q

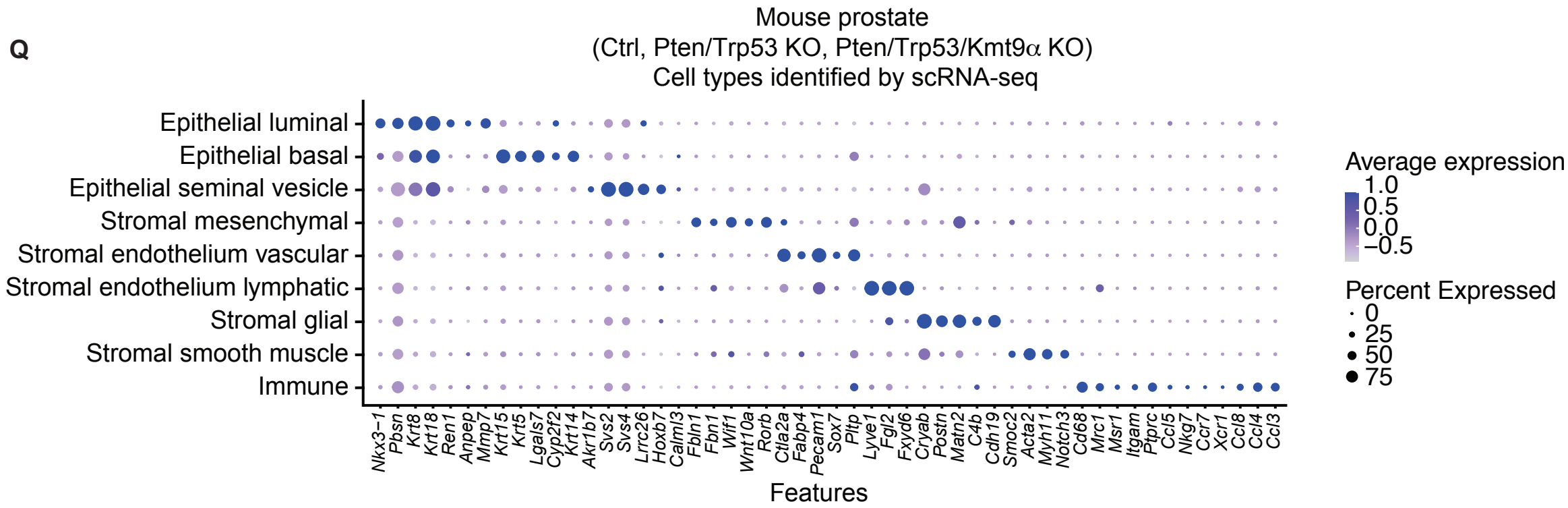

R

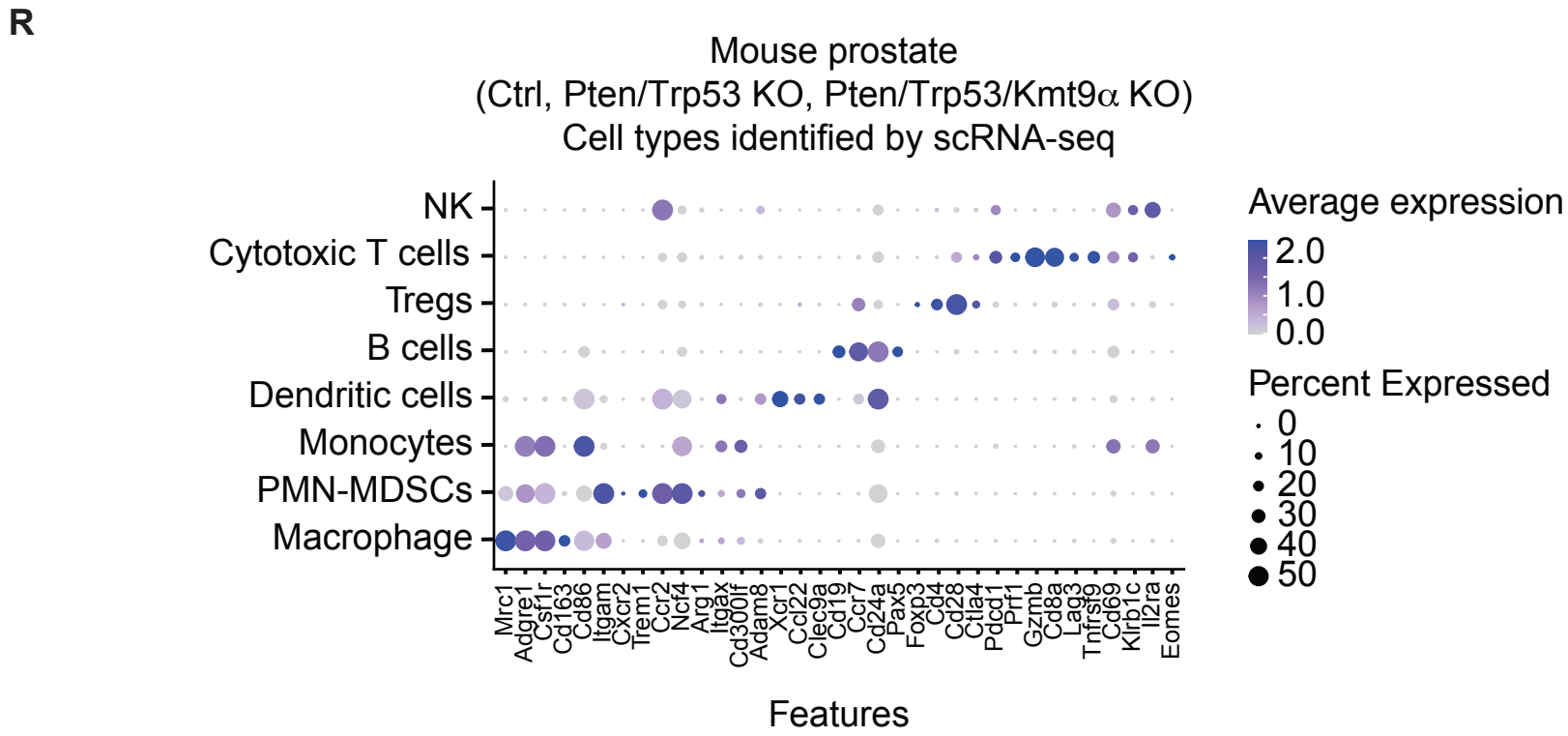

Supplemental Fig. 1: KMT9 controls expression of PMN-MDSC-chemoattractants in prostate cancer

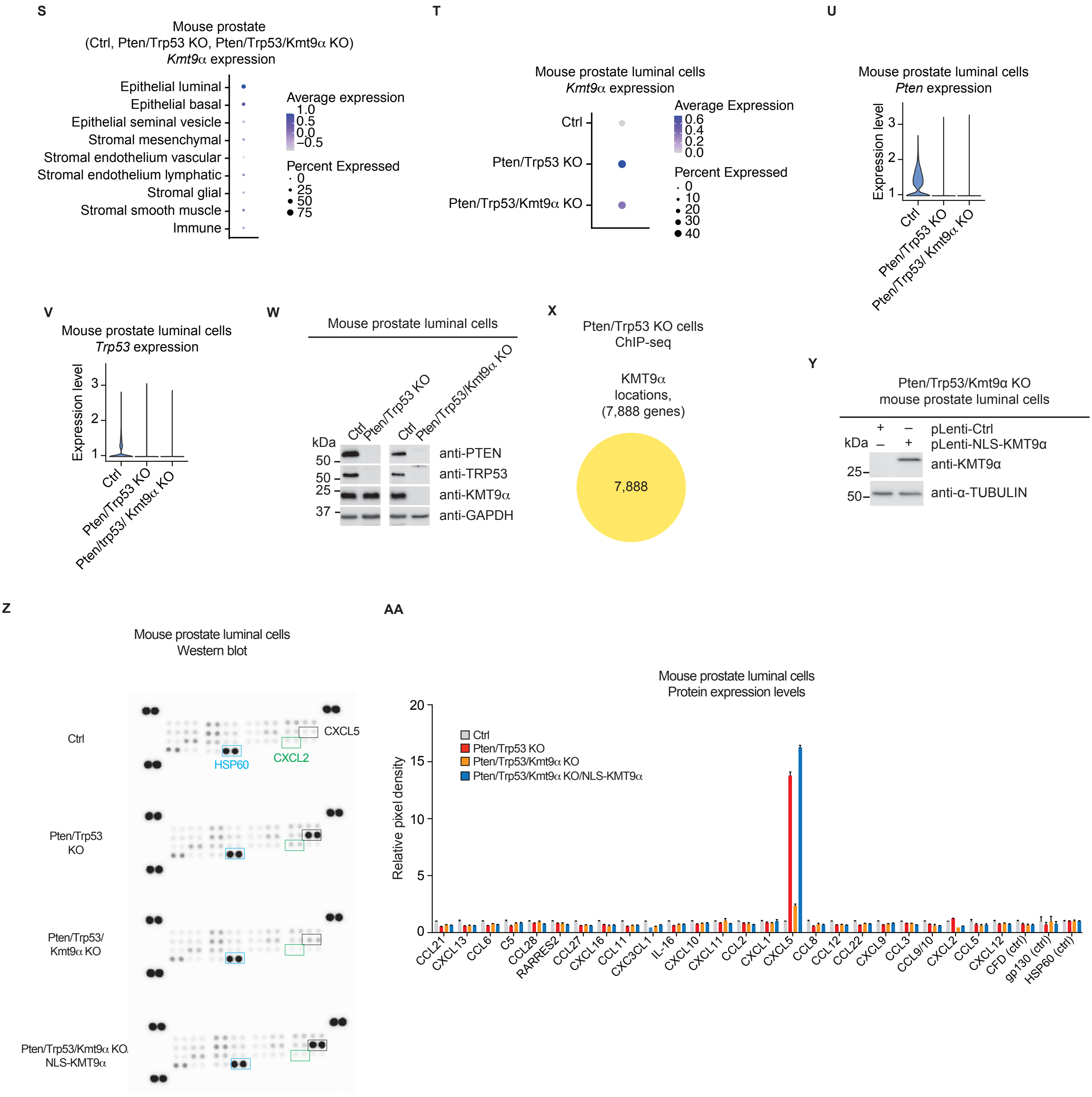

Supplemental Fig. 2: KMT9 regulates composition and spatial distribution of immune cell populations within the TIME

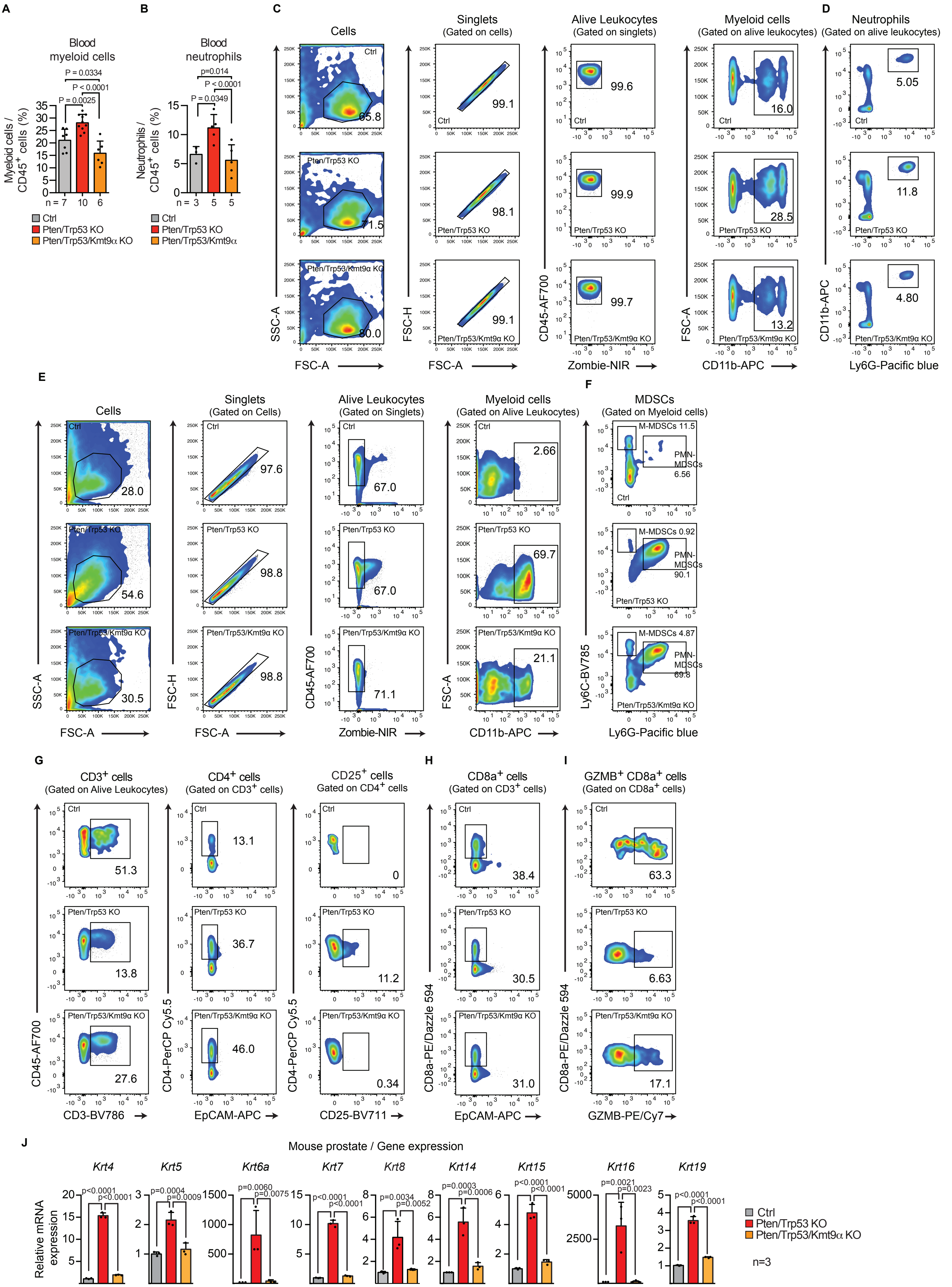

Supplemental Fig. 2: KMT9 regulates composition and spatial distribution of immune cell populations within the TIME

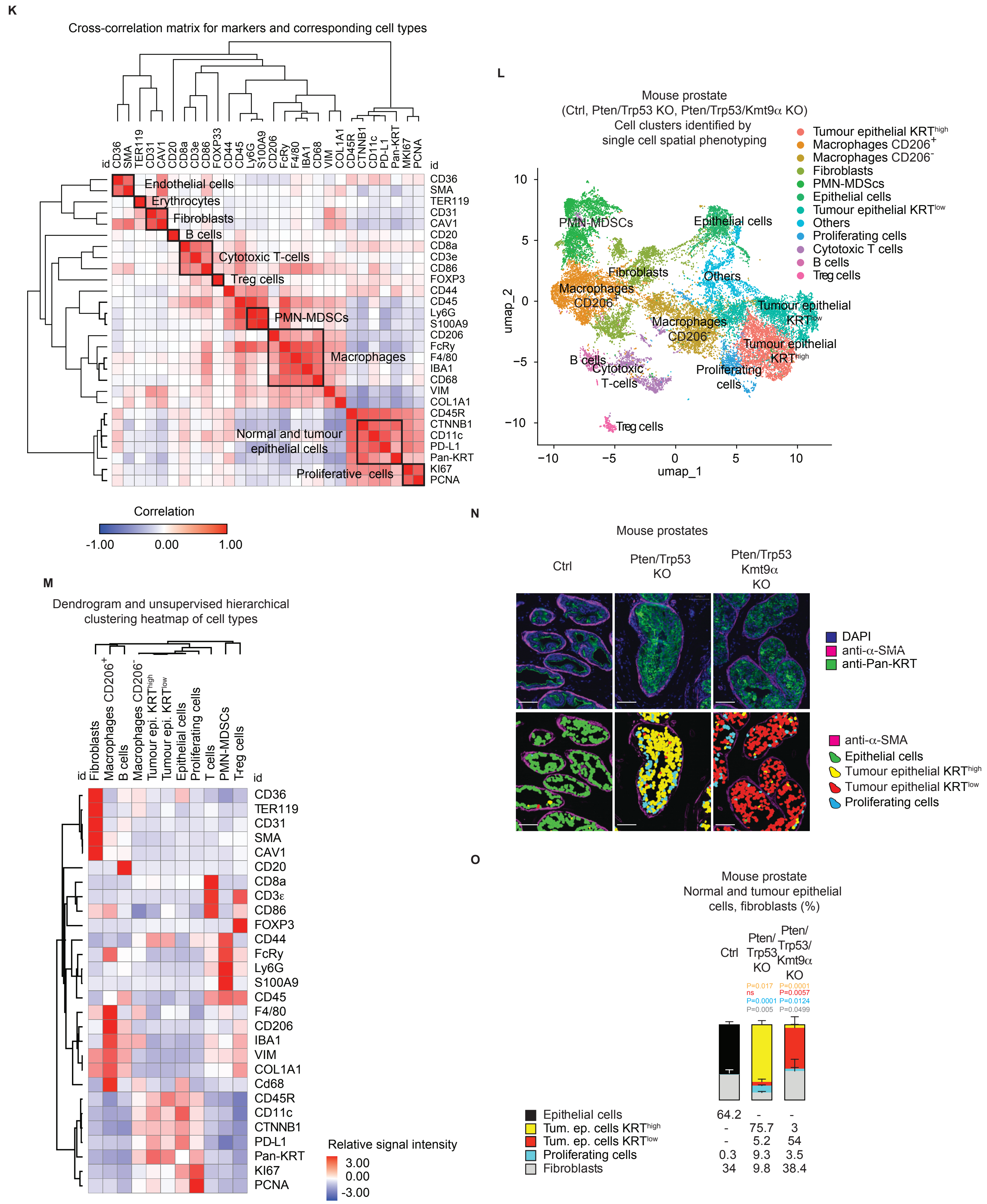

Supplemental Fig. 3: *Kmt9α* loss combined with CXCR2 inhibition results in increased inhibition of prostate tumour growth

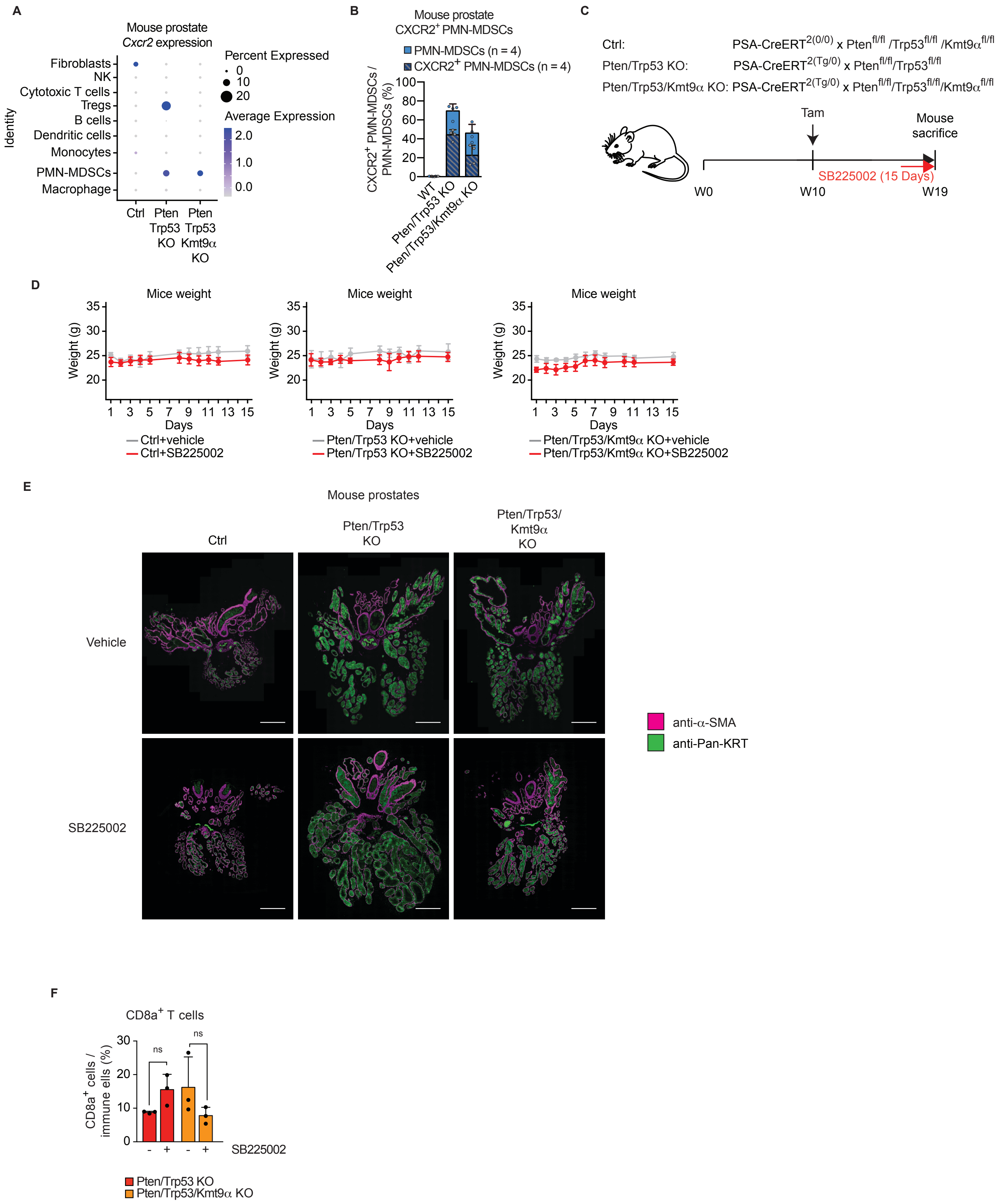

Supplemental Fig. 4: KMT9-regulated expression of ARG1 impairs T cell cytotoxicity in prostate cancer.

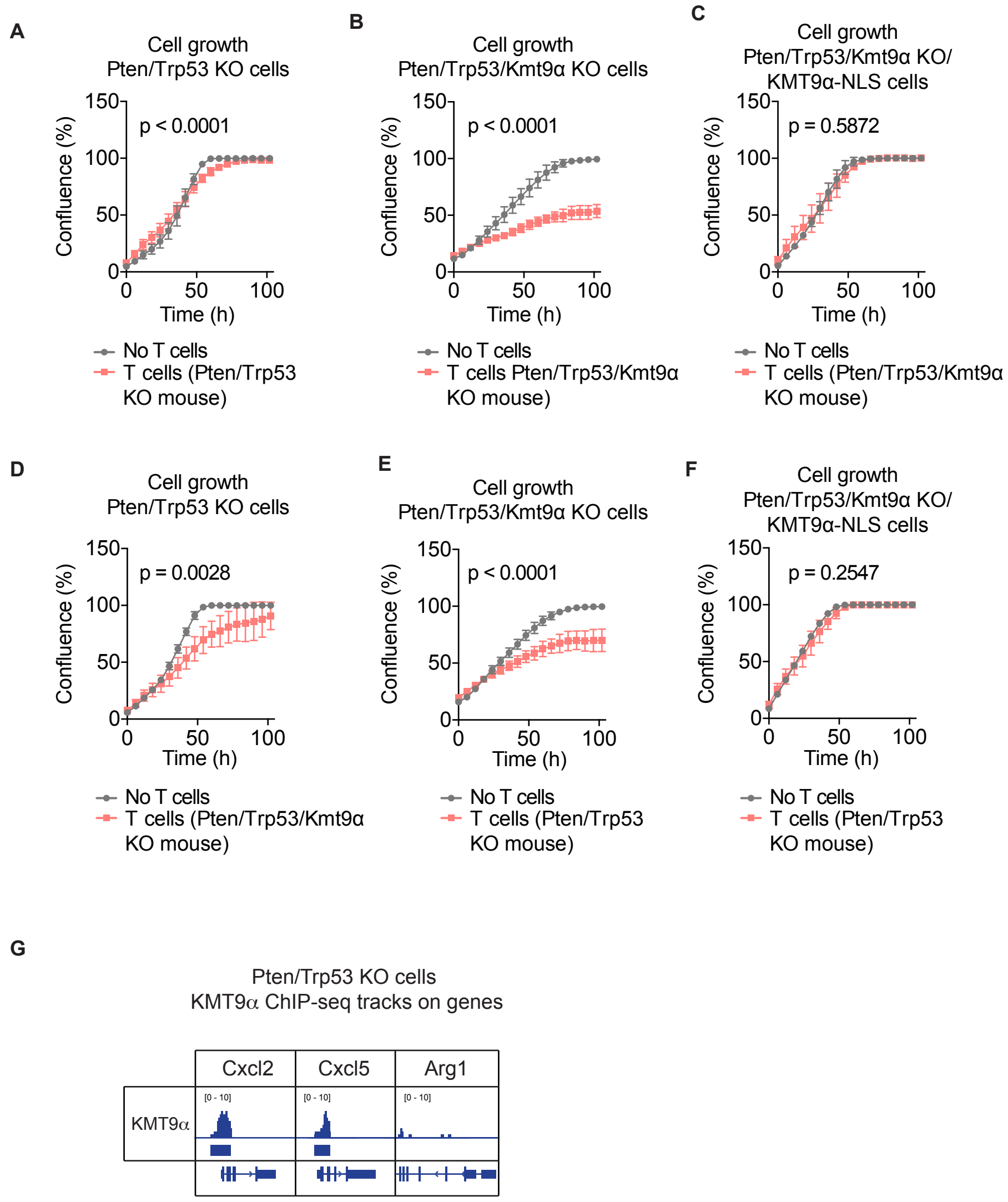

Supplemental Fig. 5: *Kmt9α* loss combined with ARG1 inhibition induces increased inhibition of prostate tumour growth

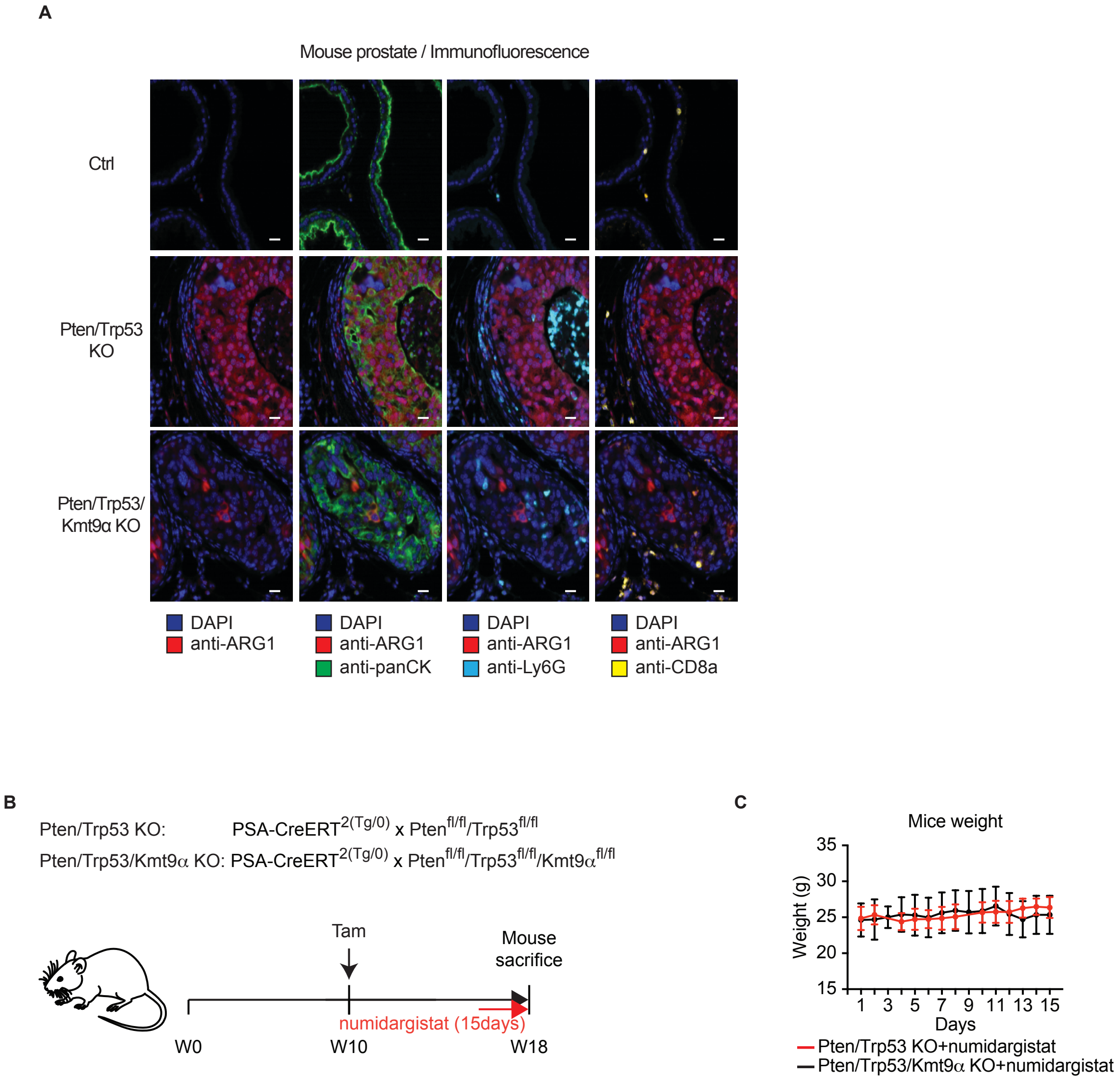

Supplement: Supplementary file 1 [file 12943_2026_2801_MOESM1_ESM.pdf]
