# Supplementary material for: KMT9 drives T cell exclusion and dysfunction by promoting PMN-MDSCs infiltration and ARG1 expression in prostate cancer
Source: Mol Cancer. 2026 Sep 17;25:223. doi: 10.1186/s12943-026-02801-8 (PMC13587405; doi:10.1186/s12943-026-02801-8)
Supplement: Supplementary file 2 [file 12943_2026_2801_MOESM2_ESM.pdf]

Supplemental Figure S6: Source data for Western blots

Figure 5F

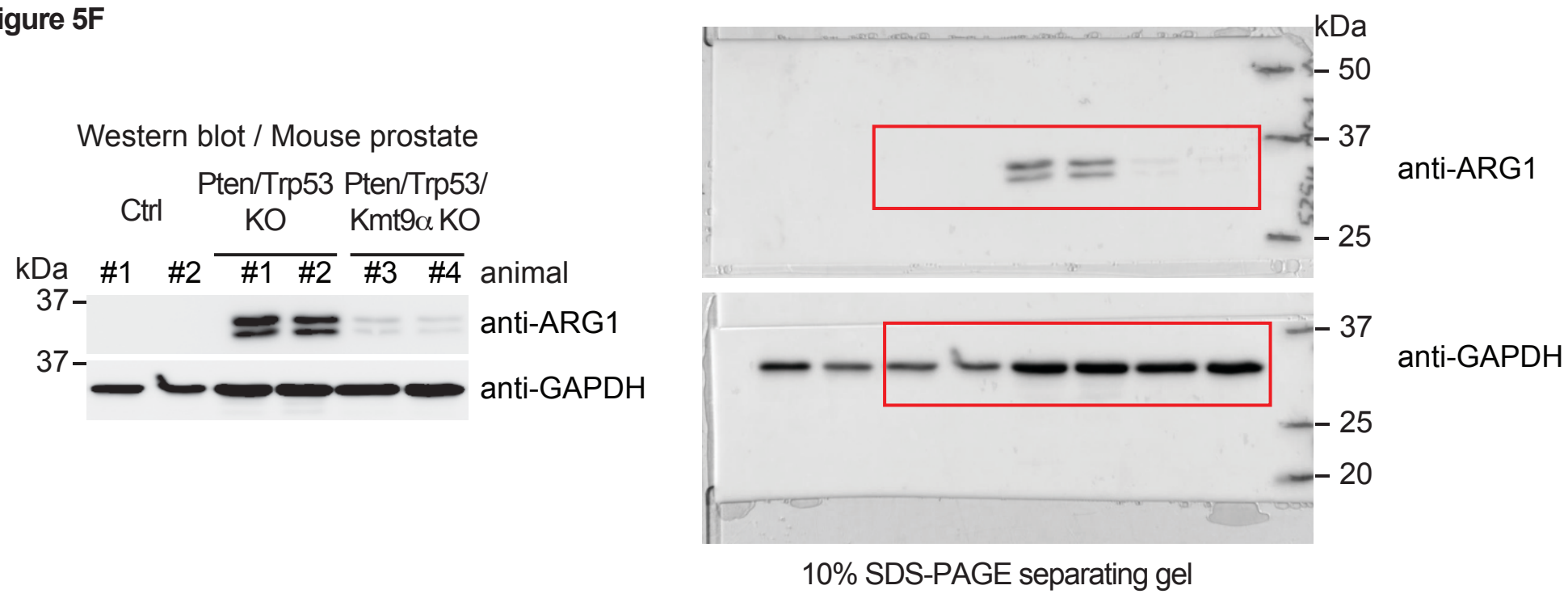

Supplemental Fig. 1H

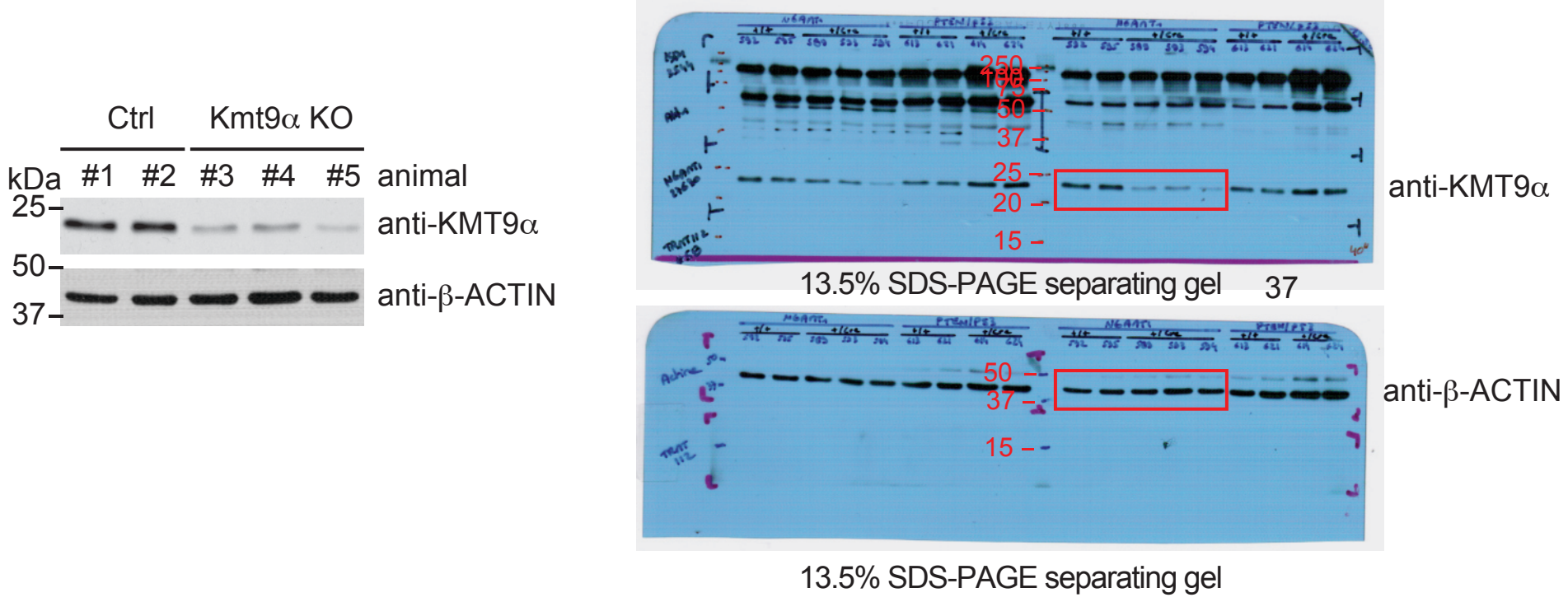

Supplemental Fig. 1I

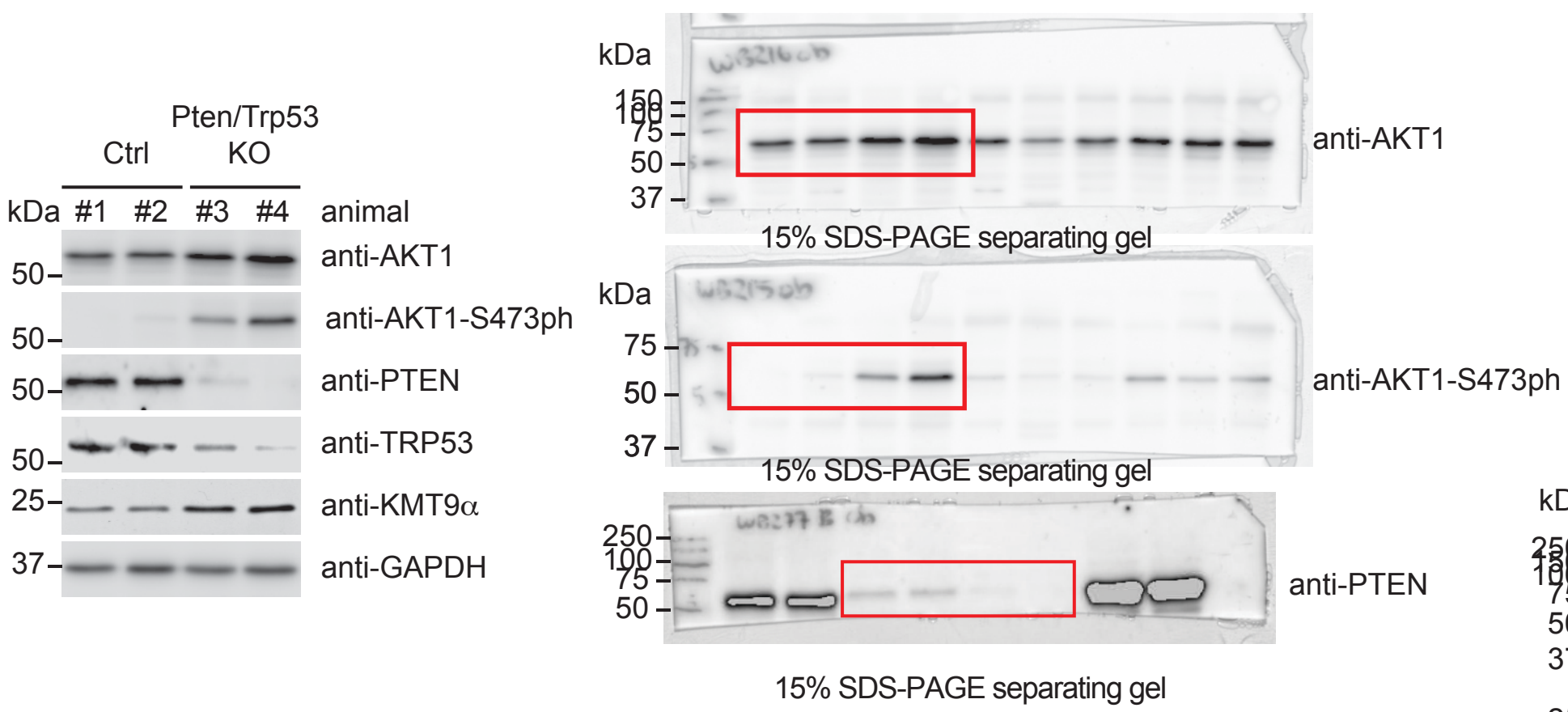

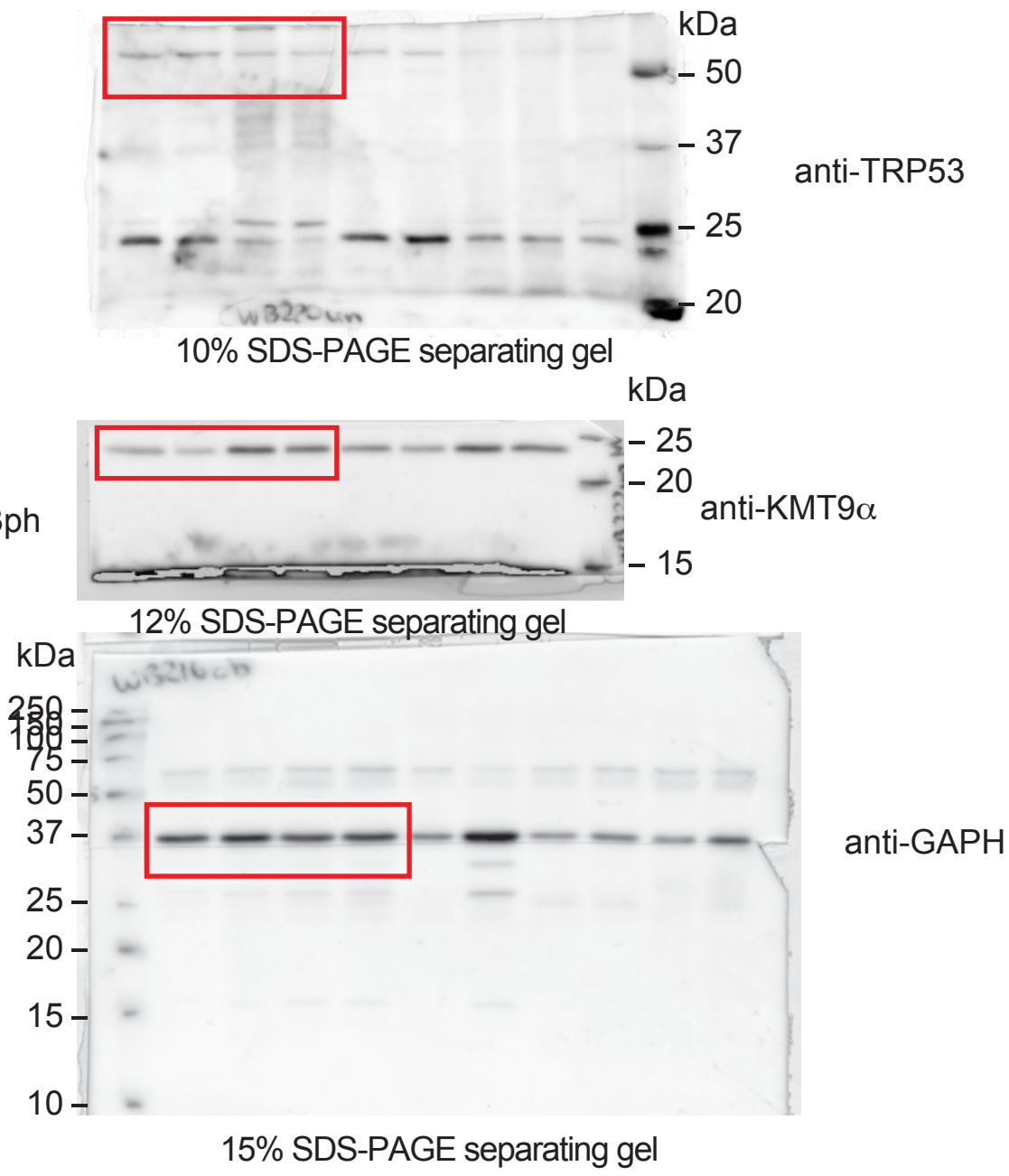

Supplemental Fig. 1J

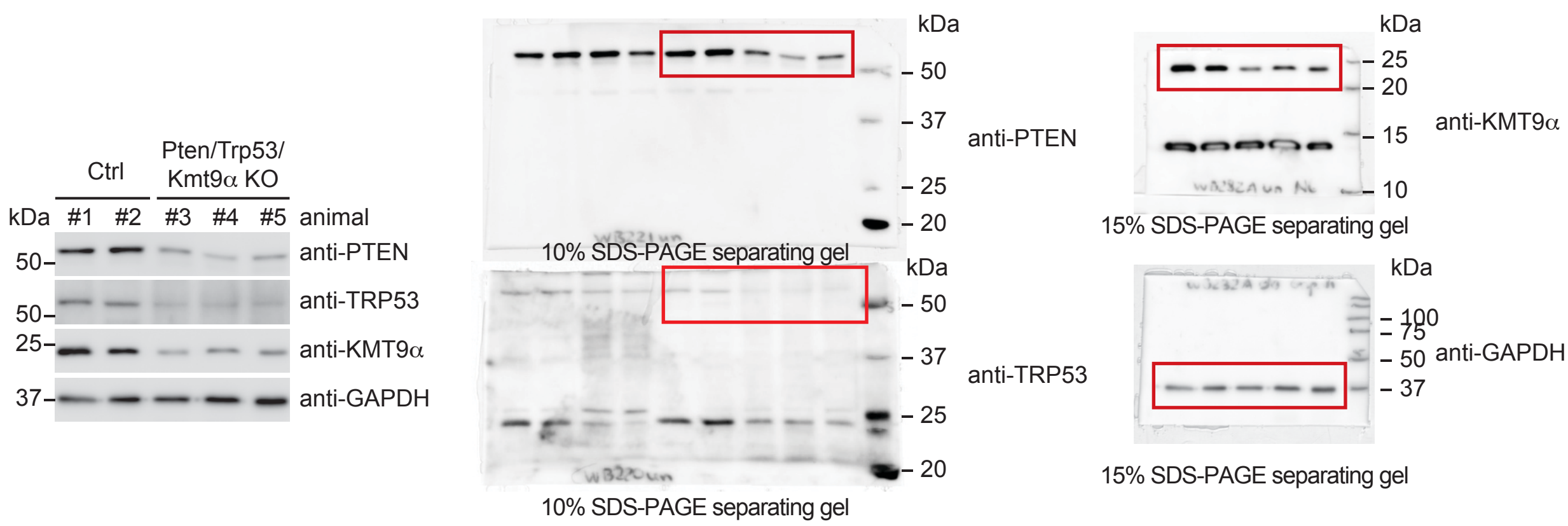

Supplemental Fig. 1W

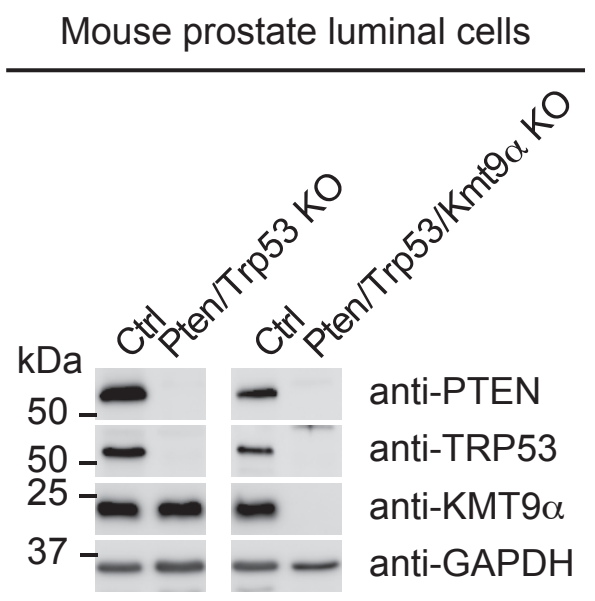

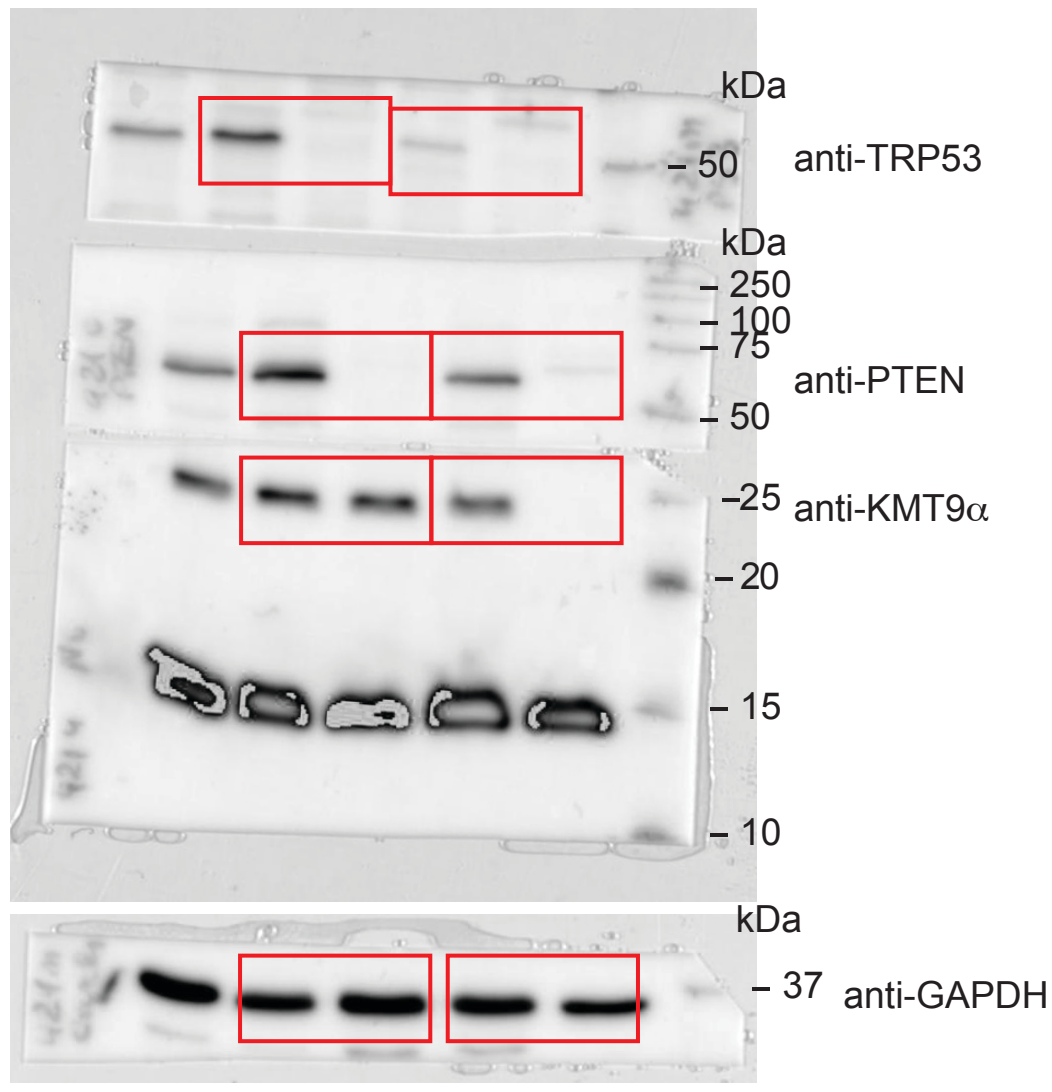

15% SDS-PAGE separating gel

Supplemental Fig. 1Y

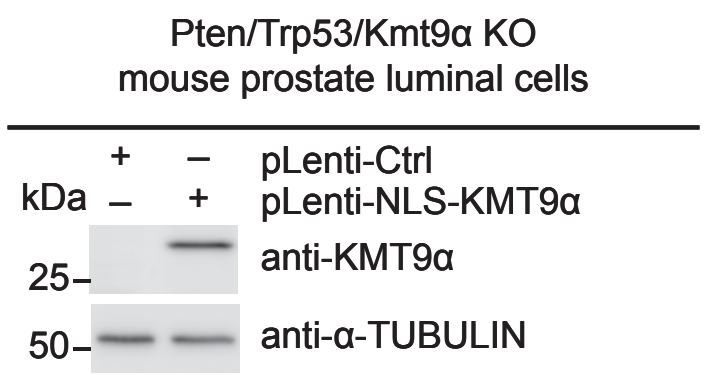

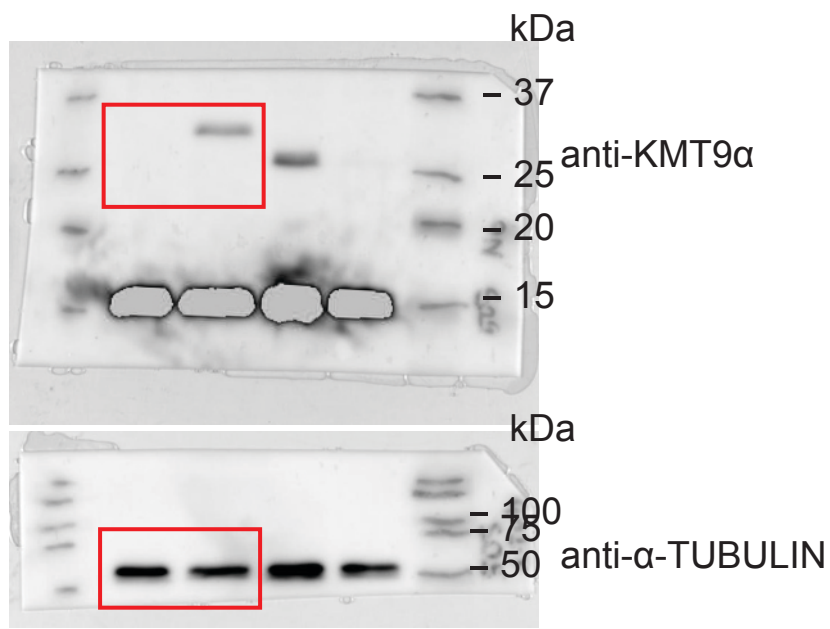

15% SDS-PAGE separating gel
